# Brazilian Society of Otology task force – Otosclerosis: evaluation and treatment

**DOI:** 10.1016/j.bjorl.2023.101303

**Published:** 2023-08-17

**Authors:** Vagner Antonio Rodrigues Silva, Henrique Furlan Pauna, Joel Lavinsky, Guilherme Corrêa Guimarães, Nicolau Moreira Abrahão, Eduardo Tanaka Massuda, Melissa Ferreira Vianna, Cláudio Márcio Yudi Ikino, Vanessa Mazanek Santos, José Fernando Polanski, Maurício Noschang Lopes da Silva, André Luiz Lopes Sampaio, Raul Vitor Rossi Zanini, Luiz Fernando Manzoni Lourençone, Mariana Moreira de Castro Denaro, Daniela Bortoloti Calil, Carlos Takahiro Chone, Arthur Menino Castilho

**Affiliations:** a Universidade Estadual de Campinas (Unicamp), Faculdade de Ciências Médicas (FCM), Departamento de Otorrinolaringologia, Cirurgia de Cabeça e Pescoço, Campinas, SP, Brazil; b Hospital Universitário Cajuru, Departamento de Otorrinolaringologia, Curitiba, PR, Brazil; c Universidade Federal do Rio Grande do Sul (UFRGS), Departamento de Ciências Morfológicas, Porto Alegre, RS, Brazil; d Universidade de São Paulo (USP), Faculdade de Medicina de Ribeirão Preto, Departamento de Oftalmologia, Otorrinolaringologia e Cirurgia de Cabeça e Pescoço, Ribeirão Preto, SP, Brazil; e Irmandade Santa Casa de Misericordia de São Paulo, Departamento de Otorrinolaringologia, São Paulo, SP, Brazil; f Universidade Federal de Santa Catarina, Departamento de Cirurgia e Hospital Universitário, Florianópolis, SC, Brazil; g Universidade Federal do Paraná, Hospital de Clínicas, Departamento de Otorrinolaringologia e Cirurgia de Cabeça e Pescoço, Curitiba, PR, Brazil; h Faculdade Evangélica Mackensie do Paraná, Curitiba, PR, Brazil; i Universidade Federal do Rio Grande do Sul (UFRGS), Departamento de Otorrinolaringologia, Porto Alegre, RS, Brazil; j Universidade de Brasília (UnB), Faculdade de Medicina, Laboratório de Ensino e Pesquisa em Otorrinolaringologia, Brasília, DF, Brazil; k Hospital Israelita Albert Einstein, São Paulo, SP, Brazil; l Universidade de São Paulo, Faculdade de Odontologia de Bauru, Bauru, SP, Brazil; m Universidade de São Paulo, Hospital de Reabilitação de Anomalias Craniofaciais, Bauru, SP, Brazil; n Universidade Federal de Minas Gerais (UFMG), Hospital das Clínicas, Belo Horizonte, MG, Brazil

**Keywords:** Hearing loss, conductive, Hearing loss, mixed conductive-sensorineural, Otosclerosis, Stapes surgery

## Abstract

**Objectives:**

To review and provide evidence-based recommendations for the diagnosis and treatment of otosclerosis.

**Methods:**

Task force members were educated on knowledge synthesis methods, including electronic database search, review and selection of relevant citations, and critical appraisal of selected studies. Articles written in English or Portuguese on otosclerosis were eligible for inclusion. The American College of Physicians’ guideline grading system and the American Thyroid Association’s guideline criteria were used for critical appraisal of evidence and recommendations for therapeutic interventions.

**Results:**

The topics were divided into 2 parts: 1) Diagnosis – audiologic and radiologic; 2) Treatment – hearing AIDS, pharmacological therapy, stapes surgery, and implantable devices – bone-anchored devices, active middle ear implants, and Cochlear Implants (CI).

**Conclusions:**

The pathophysiology of otosclerosis has not yet been fully elucidated, but environmental factors and unidentified genes are likely to play a significant role in it. Women with otosclerosis are not at increased risk of worsening clinical condition due to the use of contraceptives or during pregnancy. Drug treatment has shown little benefit. If the patient does not want to undergo stapedotomy, the use of hearing aids is well indicated. Implantable systems should be indicated only in rare cases, and the CI should be indicated in cases of profound deafness.

## Highlights

• There is no evidence that pregnancy increases the risk of developing or worsening otosclerosis.
• The use of the endoscope in stapes surgery is equally as safe as the use of the microscope.
• No prosthesis material is superior to another in stapedotomy regarding hearing outcomes.
• Among nonsurgical treatment options, hearing devices provide the best result.

## Introduction

Otosclerosis is a disease characterized by abnormal remodeling in the otic capsule.^1^ Bone remodeling is a natural process that is ongoing throughout the skeleton, consisting of a balance between bone resorption by osteoclasts and bone formation by osteoblasts.^2^ Otosclerosis only affects the temporal bone, particularly the *fissula ante fenestram*, but may extend to the region of the labyrinth and cochlea, oval window, and round window. Histopathologic characteristics include focal osteolytic bone lesions with increased cellularity and vascularity.^3^

Mean age at onset ranges from 15 to 45 years, and women are 2–3 times more affected than men. Approximately 60% of patients with clinical otosclerosis have a family history of the disease. The remaining 40% is thought to represent autosomal dominant hereditary cases with failed penetrance, new mutations, viruses, environmental etiology, or rare cases of autosomal recessive inheritance.

The classic presentation of otosclerosis consists of progressive conductive hearing loss in adulthood. However, the type of deafness depends on the location and extension of the otosclerotic *foci*. Lesions that originate in the *fissula ante fenestram* and involve the annular ligament cause conductive deafness, whereas medial progression to the cochlear endosteum causes sensorineural deafness. Tinnitus is a highly prevalent symptom. Patients may describe improved hearing clarity in noisy environments. This phenomenon is known as Paracusis of Willis, in which the conductive hearing loss subdues the background noise such that it improves the signal-to-noise ratio for the patient.^1^

Vestibular symptoms have been reported in up to 40% of patients with otosclerosis. Vestibular complaints should be investigated during clinical evaluation, as misdiagnosis can have significant implications on treatment outcomes, especially in patients with Ménière’s disease, an enlarged vestibular aqueduct, or superior semicircular canal dehiscence. A case-control study^4^ found an association between otosclerosis and osteoporosis when compared with controls with presbycusis (OR = 4.64; 95% CI 1.35–9.79).

Patients with otosclerosis commonly present with normal otoscopy. Hyperemia may sometimes be observed on the cochlear promontory and is characterized by anastomoses between the otosclerotic *foci* (with superficial venous lakes) and vessels of the cochlear promontory submucosa, which can be seen through the tympanic membrane. This is known as the Schwartze sign; it was first described in 1873 and represents the active phase of the disease.^5^ This sign is inconsistently found in patients with otosclerosis and is not necessary for diagnosis.^6^

Examination using 256 Hz and 512 Hz tuning forks is important to confirm audiometric results and assess the indication for surgery. If the examination differs from the audiogram, the audiogram should be repeated. In the Weber test, the patient will perceive sound in the ear with conductive loss or, in bilateral cases, the ear with greater hearing loss. This test is sensitive to a 5 dB difference between ears. The Rinne test is negative when sound conducted via the bone of the mastoid process is heard louder by the patient than airconducted sound, suggesting conductive hearing loss. The 256 Hz tuning fork is sensitive to a 10–15 dB Air-Bone Gap (ABG), whereas the 512 Hz tuning fork is sensitive to a 20‒5 dB ABG.^7^ These tests should not replace formal audiometric tests in patients with suspected otosclerosis or other disorders.

### Epidemiology

Otosclerosis is more commonly found in Caucasian patients, among whom 1% may present symptoms. Some temporal bone series reported histologic evidence of otosclerosis in up to 10% of cases, of which only 12% developed the clinical form. The incidence of otosclerosis is lower in Asian patients^8^, ^9^ and even rarer in Black African patients.^10^ A study conducted in Houston, TX, USA, found an overall prevalence of 20 cases of otosclerosis per 100,000 patients in the health system. Most patients were Hispanic (43/100,000), followed by Caucasian (12.6/100,000) and African American patients (3/100,000).^11^ Although the prevalence of histologic changes in Japanese patients is the same as in Caucasian patients, the otosclerotic *foci* were less extensive, did not involve the anterior site to the oval window as much, and had low activity.^8^ Otosclerosis rarely affects children, occurring in 0.6% of the population before the age of 5 and in 4% between the ages of 5 and 18.^12^

The incidence of otosclerosis increased rapidly throughout the 1960s,^13^, ^14^ but reports emerged in the late 1970s suggesting that it was decreasing.^15^ In the following decades, several studies reported that the number of stapedectomy cases had declined over the past years, which also confirmed the decline in the incidence of otosclerosis.^15^, ^16^, ^17^ The current incidence of otosclerosis is believed to be lower than it was 50 years ago.^18^ A large US population study (Rochester Epidemiology Project) assessed the incidence of otosclerosis between 1950 and 2017. The incidence was originally 8.9 cases/100,000 person-years in the 1950s; it increased significantly to 18.5/100,000 in the 1970s but decreased to 6.2/100,000 in the 1990s. Between 2015 and 2017, the incidence further decreased to 3.2/100,00 person-years. This progressive decline may be a result of mass measles vaccination in the US.^18^

### Genetics

Otosclerosis can affect more than one person in the same family but can also affect patients with no family history of the disease. In affected families, otosclerosis may be monogenic, meaning that one mutation is sufficient to cause the disease. In sporadic cases, a complex genetic form may be involved, in which the disease is probably caused by a combination of multiple genetic and environmental factors.^19^

Approximately 50%–60% of patients with otosclerosis have a positive family history.^20^ In most families, the inheritance pattern is autosomal dominant with incomplete penetrance.^1^ However, other inheritance patterns have also been proposed, such as digenic recessive, autosomal recessive, and X-linked dominant inheritance.^19^ Despite evidence of a genetic contribution to otosclerosis, the heritability of the disease has not been estimated.^19^, ^21^

In most families, otosclerosis appears to be caused by a small number of genetic factors (oligogenic), while in only a small number of families the disease seems to be truly monogenic. In the remaining patients, a complex genetic form of otosclerosis is present. Several studies have identified underlying genetic factors, which have led to the identification of 8 published *loci* for monogenic Otosclerosis (OTSC), as well as several genes and a chromosomal region (11q13.1) with a clear association with the disease. The implementation of next-generation sequencing in otosclerosis research has led to the identification of pathogenic variants in the MEPE, ACAN, and SERPINF1 genes, although the pathogenic role of the latter is still under debate. Furthermore, a recent genome‑wide association study can be considered a breakthrough for otosclerosis, as it identified several strong associations and suggested new potential candidate genes. These recent findings are important to unravel the genetic architecture of the disease, but further studies are needed to help understand its complete pathogenesis.^19^

Genetic studies of families with several affected members investigated the location of the involved gene in chromosomes using linkage analysis. Eight different *loci* for otosclerosis have been identified to date: OTSC1 (position 15q25−26)^22^; OTSC2 (position 7q34−36)^23^; OTSC3 (position 6p21.3–22.3)^24^; OTSC4 (position 16q21−23.2)^25^; OTSC5 (position 3q22−24)^26^; OTSC7 (position 6q13−16.1)^27^; OTSC8 (position 9p13.1-q21.11)^28^; OTSC10 (position 1q41−44).^29^ OTSC6 findings have not yet been published. However, precise identification of the genes involved in the manifestation of otosclerosis is yet to be achieved. An exception would be the OTSC2 *locus*, where a lower expression of T-cell receptor-β was observed in the peripheral blood mononuclear cells of the family members being studied. In this case, there would be changes in the development and aging of T-cells in these patients, but the events that would lead to abnormal bone remodeling were not elucidated.^30^

The genetic variants involved in complex inheritance are different from those involved in monogenic forms of the disease. Unlike variants associated with single-gene conditions, variants involved in complex diseases are neither necessary nor sufficient to cause the disease. Therefore, genetic identification is performed through association studies with a case-control design to identify variants that are significantly more frequent in patients than in controls, which would indicate that a given gene plays a role in the pathogenesis of a given disease. Association studies have been conducted with predetermined genes. Candidate gnes were selected based on the functional characteristics of a given gene. Some functional candidate genes, such as NOG, SLC26A2, POU3F4, SLAMF1, PTHR1, and COL1A2,^31^, ^32^, ^33^ have never been associated with otosclerosis. Other genes have shown association in 1 or more studies.

#### COL1A1

COL1A1 gene variants were the first to be associated with otosclerosis by McKenna et al.^31^ COL1A1 is involved in bone metabolism and is known to be associated with osteogenesis imperfecta and osteoporosis.^31^ Chen et al. identified five variants in COL1A1, as well as two haplotypes associated with otosclerosis.^33^ Other genes involved in the metabolism and chondrogenesis of the otic capsule were also investigated, such as FGF2, RARA, OTOR, and PTH, but most of them did not show an association with otosclerosis. Thus, although studies have been conducted with different populations, the results are not very reproducible, and there is limited consistent evidence supporting the association between these genes and otosclerosis.^19^

#### TNFRSF11B

The TNFRSF11B gene encodes Osteoprotegerin (OPG), a decoy receptor to activate the Receptor Activator of Nuclear Factor Kappa B Ligand (RANKL). RANKL binds to both RANK, leading to osteoclast maturation and bone resorption, and OPG, which regulates this process.^34^ Functional studies on OPG have shown that it plays a role in otosclerosis. Compared with normal stapes tissue samples, the mRNA expression of OPG is reduced in patients with otosclerosis.^35^, ^36^ In addition, homozygous mutations in TNFRSF11B play a role in Paget’s disease, which may also lead to hearing loss,^37^ making it an interesting candidate gene for otosclerosis.

#### TGFB1

The TGFB1 gene plays an important role in the development and regulation of bones and cartilage^38^ and is related to otic capsule metabolism. It has been associated with otosclerosis in two different populations.^27^ An amino acid variant at position 263 of TGFB1 (I263) was shown to be protective, suggesting that it decreases otosclerosis susceptibility. An increase in nonsynonymous variants in the TGFB1 gene was identified in patients with otosclerosis.^27^ Bone morphogenetic proteins 2 and 4 (BMP2 and BMP4), which are members of the TGFB superfamily and play important roles in several stages of bone metabolism, have also been associated with otosclerosis susceptibility.^39^ A study investigating rare and common variations in BMP2 and BMP4 did not identify an association between common variants and otosclerosis. However, 4 rare variations were identified, and the functional analysis showed a reduction in phosphorylation of the receptor Smad.^40^ These results suggest that BMP2 and BMP4 play a role in the pathophysiology of otosclerosis.^19^

### Environmental factors

In the absence of a positive family history (which accounts for almost half of cases of otosclerosis), the disease behaves in a complex way and is caused by a combination of environmental and genetic risk factors. The genetic factors that play a role in the development of otosclerosis are involved in several molecular pathways, including bone remodeling, immune pathways, inflammation, and endocrine pathways.^21^ Several environmental factors have been described, such as sodium fluoride, endocrine factors, and measles virus infection.^1^, ^21^

Fluoride ingestion may influence the prevalence of diseases with abnormal bone resorption. An epidemiological study on otosclerosis and fluoridated drinking water showed a higher prevalence of clinical otosclerosis in low-fluoride areas.^41^ Sodium fluoride neutralizes proteolytic enzymes that can cause abnormal bone metabolism, such as the Diastrophic Dysplasia Sulfate Transporter (DTDST, or SLC26A2).^42^, ^43^, ^44^

### Measles virus and otosclerosis

Measles is an RNA virus that belongs to the *Paramyxoviridae* family. It is a highly contagious viral disease that clinically presents with fever, malaise, rash, cough, runny nose, and conjunctivitis. Mass vaccination against measles has reduced its incidence, morbidity, and mortality.^45^ Complications include neurological disorders such as acute disseminated encephalomyelitis, measles inclusion body encephalitis, and subacute panencephalitis. Other complications are keratoconjunctivitis, stomatitis, laryngitis, diarrhea, pneumonia, and otitis media. Measles can also complicate pregnancy and lead to adverse outcomes. It can affect multiple organ systems and may lead to death.^45^

The measles virus may be related to the etiopathogenesis of otosclerosis. This hypothesis is reinforced by the decline in otosclerosis prevalence after the introduction of measles vaccination.^46^ Most observational studies detected measles virus RNA in stapes of patients with otosclerosis using different methods. Elevated levels of measles virus-specific immunoglobulin G are found in the perilymph of patients with otosclerosis.^47^ Several observational studies have used methodologies such as reverse transcription polymerase chain reaction, quantitative reverse polymerase chain reaction, and glyceraldehyde 3-phosphate to detect measles in stapes samples from patients with otosclerosis and controls.^48^, ^49^ Liktor et al.^50^ associated the presence of measles virus with TGFB1.

Karosi et al.^51^ and Niedermeyer et al.^49^ detected measles virus mRNA in most stapes of patients with otosclerosis in several studies evaluating thousands of patients,^46^, ^52^ indicating that this virus may play a role in the pathophysiology of the disease. Arnold et al.^46^ and McKenna et al.^53^ also detected measles virus RNA, its antigens, or antibodies in a high number of samples from patients with otosclerosis.^53^, ^54^, ^55^ There was also a decline in the incidence of otosclerosis and a change in the age distribution to the population with more than 54 years of age. This was largely due to widespread measles vaccination, as reported in some European studies.^46^, ^52^

Other studies have failed to find an association between measles virus infection and otosclerosis.^56^ Singh et al.^57^ detected Immunoglobulin M (IgM) antibodies against measles in 18.1% of participants and IgM antibodies against varicella zoster virus in 4.5%,^57^ concluding that otosclerosis is not associated with a systemic viral measles infection. Flores-García ML et al.^58^ conducted an observational study and detected measles virus mRNA in only 3.3% (3 out of 93) of participants. Komune et al.^48^ and Grayeli et al.^59^ also failed to detect the presence of measles virus infection in most of their study sample.^48^, ^59^ However, the samples were smaller, and the authors used different detection methods.

### The influence of female hormones on the progression of otosclerosis

Sex steroid hormones play an important role in the regulation of bone metabolism. (Imauchi, 2004, Effect of 17 beta-estradiol on diastrophic dysplasia sulfate transporter activity in otosclerotic bone cell cultures and SaOS-2 cells). Estrogen has been implicated in the development of otosclerosis because women are affected more often than men and because the disease often manifests or progresses during or shortly after pregnancy. Estrogen receptors can be found on otosclerotic cells, although the regulatory mechanisms related to these receptors is unknown.^60^ Estrogen has an established role in osteoblastic function, the role of osteoblasts in otosclerosis is unclear, and no sex hormone has been directly implicated in otosclerosis. Although there are reports of hearing loss related to hormone replacement therapy and oral contraception, in a large cohort of approximately 17,000 women followed up for up to 26 years, no association was found between the use of oral contraceptives and the development of otosclerosis.^61^, ^62^ Lippy et al.^63^ conducted a retrospective study with 94 women with otosclerosis, divided into two groups (with vs without children), and found no adverse effects on hearing in women who had children compared with those without children, even with the increasing number of pregnancies.

In a retrospective study of 6025 adults (3553 women and 2472 men) undergoing stapedotomy, the average age at the time of surgery was significantly lower in women than in men (46.8 vs. 48.1 years). However, both women and men with children were significantly younger at the time of surgery compared with women and men without children. The authors concluded that neither pregnancy nor the number of children influence indication for surgery.^64^

Therefore, believing that estrogen may have deleterious effects in patients with otosclerosis is counterintuitive, as several studies have shown that this hormone has a protective effect on the inner ear^65^, ^66^, ^67^: 1) It increases the expression of the antioxidant genes Superoxide Dismutase (SOD), thereby reducing ROS-induced apoptosis in Hair Cells (HCs); 2) It directly upregulates anti-apoptotic genes such as Bcl-2 and Bcl-X_L_ and could be involved in the protection and survival of HCs and spiral ganglion nerves; 3) It upregulates neuroglobin, a potent ROS scavenger that mediates a vasorelaxant effect that can improve inner ear and stria vascularis perfusion, preserving HCs; 4) It regulates many ion channels, including K^+^ channels expressed in strial cells that are crucial for endolymph composition and mechanotransduction; and 5) It could reduce cochlear inflammation by inhibiting NLRP3 expression or activation in cochlear resident macrophage-like cells and the release of pro-inflammatory cytokines.

### Otopathology

Otosclerosis may be classified according to clinical presentation or histopathologic findings (Box 1).

**Box 1:** Otosclerosis clinical presentation and histopathologic findings.

Histologic otosclerosis is limited to the otic capsule and refers to cases without footplate fixation or clinical repercussions, therefore it is an accidental finding on temporal bone autopsies.^5^, ^68^
Clinical otosclerosis is characterized by a lesion that fixes the stapes footplate in association with auditory and vestibular symptoms (hearing loss, tinnitus, vertigo).^69^
Cochlear otosclerosis refers to invasion of the cochlear endosteum with extensive involvement of the otic capsule, without stapes fixation, leading to NSHL, tinnitus, and vestibular symptoms.^69^

#### Histopathology

The ossicular chain and otic capsule undergo endochondral ossification during their development and, after this process, minimal bone remodeling occurs throughout life. Bone remodeling has reduced activity in the petrous portion of the temporal bone and is almost null near the perilymphatic space.^42^ This is explained by the presence of OPG, a mediator produced in large quantities by the spiral ligament that inhibits the recruitment, formation, and activation of osteoclasts. Therefore, low levels of OPG may be related to pathological new bone formation and resorption.^42^ Several cytokines are likely to be active in otosclerotic lesions, and the disinhibition of one or more of these cytokines may trigger the development of otosclerosis. Although other cells, such as osteocytes and bone lining cells, may contribute to calcium flux on bone surfaces, bone remodeling only occurs through the action of osteoblasts and osteoclasts.^42^

The otic capsule contains regions of immature cartilage called globuli interossei, which may correspond to the earliest *loci* of otosclerosis.^42^ The otosclerotic focus is identified on histologic sections of the temporal bone by its distinct appearance in the otic capsule after undergoing a remodeling process in which normal bone is replaced by otosclerotic bone. The otosclerosis focus may appear as dense mineralized bone (sclerosis) or active, well-vascularized bone (spongiotic).^42^ One of the first histologic manifestations of otosclerosis is known as blue mantles, which are basophilic staining regions visualized after application of Hematoxylin and Eosin (H&E). They are found near regions of otosclerosis and probably represent bone that has been recently remodeled, also known as basophilic bone.^42^

Another remarkable characteristic of the initial process of otosclerosis are the vascular channels, which result from an enlargement of the perivascular spaces. Bone is resorbed around a vessel and replaced by a fibrous connective tissue. These areas of active disease are characterized by the presence of osteoclastic giant cells and vascular proliferation. Within this space, reticular cells and fibroblasts assume the form of osteoblasts. At the same time, calcification begins in the matrix and a new, immature bone is formed with a bluish stain on H&E.^70^ Depending on whether the disease is active or inactive, it is termed otospongiosis (active) or otosclerosis (inactive).

Osteoblasts and osteoclasts precursors, histiocytes, and macrophages are commonly observed on electron microscopy. The otosclerotic process does not respect the normal limits and contours of the labyrinth or ossicles and may become exophytic and extend into the middle ear and perilymphatic space.^42^

Otosclerosis is limited to the temporal bone, and involvement of other regions has never been described.^42^ In approximately 70%–80% of patients, both temporal bones are affected by otosclerosis.^70^ *Foci* of otosclerosis consist of bone formation by osteoblasts, bone destruction by osteoclasts, vascular proliferation, and a stroma of fibroblasts and histiocytes. The main focus of otosclerosis (96%) is located anterior to the stapes footplate (*fissula ante fenestram*),^42^ but only 10%–15% of patients present stapes ankylosis.^5^, ^70^ Another commonly affected region is the round window niche (in 30%–50% of cases), but complete obliteration of the niche is rare.^5^, ^70^

*Foci* of otosclerosis can also be found posterior to the oval window, on the posterior wall of the Internal Auditory Canal (IAC), around the cochlear aqueduct, and involving the semicircular canals and leading to the thickening of the stapes footplate.^71^ Extensive involvement of the oval window and footplate may be present in 7%–11% of cases, whereas round window obliteration is found in 1%.^69^ Cases without involvement of the ossicular chain are rare.^42^ Schuknecht and Kirchner^68^ showed that when otosclerosis is severe enough to extend into the cochlear endosteum, the onset of Sensorineural Hearing Loss (SNHL) symptoms is typically associated with stapes fixation. Ankylosis results from an enlargement of the otosclerotic focus that affects the stapes footplate and then involves the cartilage at the margin of the oval window, replacing it with immature and fibrotic bone tissue that is thicker and involves the annular ligament.^70^

After the otosclerotic focus reaches the cochlear endosteum, atrophy of the stria vascularis and formation of hyalinization in the spiral ligament occur.^5^, ^68^ This process has been associated with impairment of ionic homeostasis, causing hearing impairment by reducing the cochlear potential, with subsequent HC dysfunction and leading to SNHL.^68^ Immunohistochemical staining has demonstrated that the hyalin material is composed of type I collagen, chondroitin sulfate, and keratin sulfate. In very advanced cases of otosclerosis, there may be intracochlear deposition of bone.^5^

Another characteristic of advanced otosclerosis is deformation around the cochlea, leading to an irregular appearance and narrowing of the helicotrema, as well as blockage of the cochlear and vestibular aqueducts.^5^ Otosclerosis evolves from an “otospongiotic” phase in which the normal lamellar otic capsule bone around vessels is resorbed, creating perivascular (or pseudovascular) spaces. These areas are highly cellular, with an increased number of osteoclasts. On H&E staining, these areas are often highly acidophilic, with a clear distinction between normal bone and the otosclerotic focus. Ultimately, new woven bone is deposited, which may be larger in volume than the bone that was resorbed, sometimes resulting in thickening of the involved area (e.g., the stapes footplate).

The new bone is presumably converted into lamellar bone, which is dense, and results in a highly eosinophilic and relatively acellular “sclerotic” focus.^5^ Less active otosclerotic lesions display new, woven bone formation with hypercellularity, often with more than two cells situated within a single lacunae.^42^ They represent the end stage of the disease, with bone transformation characterized by solid, lammellar, mosaic-like osseous tissue, which contains few and tiny marrow spaces as well as few and thin blood vessels. Not rarely, both inactive and active lesions can be found in a single temporal bone.^70^

**Figure 1:**
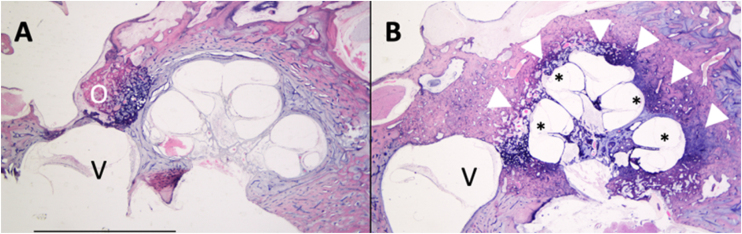
Axial section of temporal bones of patients with different stages of otosclerosis. (A) Fenestral otosclerosis. (B) Cochlear otosclerosis.; O, Otosclerotic focus on the *ante fenestram fissula*; V, Vestibule; (*), Reissner’s membrane distention compatible with endolymphatic hydrops; Arrowhead, Otosclerotic focus involving the cochlea.

Based on histologic findings that include the identification of *foci* of disordered bone resorption, new bone deposition, vascular proliferation, and/or connective tissue, 3 clinically relevant zones were defined to simplify the description of the extent of otosclerosis (Fig. 1) (Box 2).^72^

**Box 2:** Otosclerosis histologic findings.

Zone 1: the region anterior to the oval window, including the *fissula ante fenestram*.
Zone 2: the pericochlear region, which contains the otic capsule bone surrounding the cochlea.
Zone 3: the round window niche, including the round window membrane and surrounding otic capsule bone.

Several histopathologic findings are sufficient to explain and corroborate the conductive hearing loss seen in otosclerosis. However, cases with mixed or purely SNHL are not uncommon. To explain such findings, many theories have been proposed and many histopathologic studies have been conducted. In 1987, a study including 6 temporal bones with otosclerosis and purely sensorineural auditory symptoms showed a moderate reduction in ganglion cell counts within the Rosenthal’s canal, in addition to impairment of inner and outer HCs.^73^ However, the authors associated these findings with an age-related process called presbycusis and were not convinced that cochlear otosclerosis existed.^73^

Two years after the publication, other researchers analyzed a larger number of temporal bones and measured the volume of inner and outer HCs. They found that, in temporal bones with otosclerosis, there was no significant difference in counts of outer HCs and density of spiral ganglion cells between regions with and without endosteal involvement by otosclerosis. However, total outer HC counts were lower in cochleae with 2 or more sites of endosteal involvement by otosclerosis than in cochleae with 1 site of endosteal involvement.^74^ Furthermore, other studies found different degrees of degeneration of inner and outer HCs in temporal bones with otosclerosis but failed to correlate this reduction in organ of Corti cells and spiral ganglion neurons with the extent of endosteal involvement by otosclerosis.^5^

In addition to these findings, IAC diverticulum has also been found in patients with otosclerosis. In a retrospective study analyzing Computed Tomography (CT) scans and audiometry results of 807 patients, patients with otosclerosis alone were more likely to present conductive hearing loss, whereas those with otosclerosis and IAC diverticulum were more likely to present mixed hearing loss. In most patients, IAC diverticulum is an isolated finding. The authors suggested that this finding may represent a manifestation of otosclerosis in patients with SNHL alone.^75^ Another study involving 97 temporal bones demonstrated that IAC diverticula were more common in the temporal bones of patients with otosclerosis than in patients without the disease (37.5% vs. 16%; *p* = 0.019).^76^

The presence of vestibular symptoms was elucidated by a study that found a reduction in the mean density of type I HCs in the saccule of patients with otosclerosis, but only when endosteal involvement was present. (Hızlı, 2016, Quantitative assessment of vestibular otopathology in otosclerosis: A temporal bone study) In an attempt to explain the associated vestibular symptoms, it has been hypothesized that toxic metabolites may be liberated by otosclerotic *foci* into the inner ear fluids, damaging the neuroepithelium.^77^ In addition, Endolymphatic Hydrops (EH) have been observed in some patients and may also explain the presence of vestibular symptoms. EH occurs when otosclerosis involves the spiral ligament, resulting in changes in intracochlear ionic homeostasis and obstruction of the endolymphatic duct and sac.^69^ Magnetic Resonance Studies (MRI) studies have shown varying degrees of cochlear and vestibular EH often in association with symptoms of concomitant vertigo, including in patients undergoing stapedotomy.^78^ Patients with otosclerosis may present clear signs of EH, but its degree is not related to symptom intensity. By being aware of this information, surgeons might be able to predict whether patients undergoing surgery may experience symptoms similar to Ménière’s disease postoperatively, but further studies are still needed to support this hypothesis (Fig. 1).^79^

## Objective

To review and provide evidence-based recommendations for the diagnosis and treatment of otosclerosis.

## Methods

On December 8, 2022, a task force consisting of otolaryngologists, otology specialists, Brazilian Society of Otology (Sociedade Brasileira de Otologia, SBO) directors, and SBO members met (in person and remotely) to discuss the topic of this guideline. Each participant in this meeting was tasked with giving a 15 min evidence-based lecture on one of the suggested topics. After the lecture, the participants discussed the topic until reaching a consensus. Each author was asked to write a text with the current literature on the topic, based on evidence and containing the elements discussed during the meeting. A rapporteur prepared the final text, which was reviewed by 4 additional coauthors and the Brazilian Journal of Otorhinolaryngology editor.

This guideline is not intended to be a substitute for individual professional judgment. Physicians should always act and decide in a way that they believe is best for their patients, regardless of guideline recommendations. They should also operate within their scope of practice and in accordance with their training. The guidelines represent the best judgment of a team of experienced physicians addressing the scientific evidence for a given topic.

The grading system of the American College of Physicians (ACP) was used in this guideline, relating to critical appraisal and recommendations on therapeutic interventions^80^ (Table 1, Table 2). An important component of this guideline was judged to be critical appraisal of diagnostic testing studies. However, the ACP guideline grading system was not designed for this purpose.^81^, ^82^, ^83^

**Table 1:** Interpretation of the American College of Physicians’ Guideline Grading System (for Therapeutic Interventions).

Recommendation	Clarity of risk/benefit	Implications
Strong recommendation	Benefits clearly outweigh harms and burdens, or vice versa.	Patients: Most would want course of action; a person should request discussion if an intervention is not offered.
		Clinicians: Most patients should receive the recommended course of action.
		Policymakers: The recommendation can be adopted as policy in most circumstances.
Weak recommendation	Benefits closely balanced with harms and burdens.	Patients: Many would want course of action, but some may not; the decision may depend on individual circumstances.
		Clinicians: Different choices will be appropriate for different patients; the management decision should be consistent with patients’ preferences and circumstances.
		Policymakers: Policymaking will require careful consideration and stakeholder input.
No recommendation	Balance of benefits and risks cannot be determined.	Decisions based on evidence cannot be made.

**Table 2:** Recommendations (for Therapeutic Interventions) based on strength of evidence.

Recommendation and evidence of quality	Description of supporting evidence^a^	Interpretation
Strong recommendation		
High-quality evidence	RCT without important limitations or overwhelming evidence from observational studies	Can apply to most patients in most circumstances without reservation
Moderate-quality evidence	RCT with important limitations or strong evidence from observational studies	Can apply to most patients in most circumstances without reservation
Low-quality evidence	Observational studies/case studies	May change when higher-quality evidence becomes available
Weak recommendation		
High-quality evidence	RCT without important limitations or overwhelming evidence from observational studies	Best action may differ based on circumstances or patients’ values
Moderate-quality evidence	RCT with important limitations or strong evidence from observational studies	Best action may differ based on circumstances or patients’ values
Low-quality evidence	Observational studies/case studies	Other alternatives may be equally reasonable
Insufficient	Evidence is conflicting, of poor quality, or lacking	Insufficient evidence to recommend for or against

a This description of supporting evidence refers to therapy, therapeutic strategy, or prevention studies. The description of supporting evidence is different for diagnostic accuracy studies. RCT multicenter controlled trial.

The American Thyroid Association (ATA) created a diagnostic test appraisal system that used the following methodological elements: consecutive recruitment of patients representative of clinical practice, use of an appropriate reference gold standard, directness of evidence (target population of interest, testing procedures representative of clinical practice, and relevant outcomes), precision of diagnostic accuracy measures (confidence intervals for estimates such as sensitivity and specificity), and consistency of results across studies using the same test that was also used in this guideline^82^ (Table 3, Table 4).

**Table 3:** Interpretation of the American Thyroid Association Guideline for Diagnostic Tests.

Recommendation	Accuracy of diagnostic information versus risks and burden of testing	Implications
Strong recommendation	Knowledge of the diagnostic test result clearly outweighs risks and burden of testing or vice versa.	Patients: In the case of an accurate test for which benefits outweigh risks/burden, most would want the diagnostic to be offered (with appropriate counseling). A patient should request discussion of the test if it is not offered. In contrast, for a test in which risks and burden outweigh the benefits, most patients should not expect the test to be offered.
		Clinicians: In the case of an accurate test for which benefits outweigh risks/burden, most patients should be offered the diagnostic test (and provided relevant counseling). Counseling about the test should include a discussion of the risks, benefits, and uncertainties related to testing (as applicable), as well as the implications of the test result. In contrast, for a test in which risks and burden outweigh the perceived benefits, most patients should not be offered the test, or if the test is discussed, the rationale against the test should, for the particular clinical situation, be explained.
		Policymakers: In the case of an accurate test for which benefits outweigh risks/burden, availability of the diagnostic test should be adopted in health policy. In contrast, for a test in which risks and burden outweigh the perceived benefits, some restrictions on circumstances for test use may need to be considered.
Weak recommendation	Knowledge of the diagnostic test result is closely balanced with risks and burden of testing	Patients: Most would want to be informed about the diagnostic test, but some would not want to seriously consider undergoing the test; a decision may depend on the individual circumstances (eg, risk of disease, comorbidities, or other), the practice environment, feasibility of optimal execution of the test, and consideration of other available options.
		Clinicians: Different choices will be appropriate for different patients, and counseling about the test (if being considered) should include a discussion of the risks, benefits, and uncertainties related to testing (as applicable), as well as the implications of the test result. The decision to perform the test should include consideration of the patients’ values, preferences, feasibility, and the specific circumstances. Counseling the patient on why the test may be helpful or not, in her/his specific circumstance, may be highly valuable in the decision-making process.
		Policymakers: Policymaking decisions on the availability of the test will require discussion and stakeholder involvement
No recommendation	Balance of knowledge of the diagnostic test result cannot be determined.	Decisions on the use of the test based on evidence from scientific studies cannot be made.

**Table 4:** Recommendations (for diagnostic interventions) based on strength of evidence.

Recommendation and evidence of quality	Methodologic quality of supporting evidence	Interpretation
Strong recommendation		
High-quality evidence	Evidence from one or more well-designed nonrandomized diagnostic accuracy studies (i.e., observational ‒ cross-sectional or cohort) or systematic reviews/meta-analyses of such observational studies (with no concern about internal validity or external generalizability of the results)	Implies the test can be offered to most patients in most applicable circumstances
Moderate-quality evidence	Evidence from nonrandomized diagnostic accuracy studies (cross-sectional or cohort), with one or more possible limitations causing minor concern about internal validity or external generalizability of the results	Implies the test can be offered to most patients in most applicable circumstances without reservation
Low-quality evidence	Evidence from nonrandomized diagnostic accuracy studies with one or more important limitations causing serious concern about internal validity or external generalizability of the results	Implies the test can be offered to most patients in most applicable circumstances, but the utilization of the test may change when higher-quality evidence becomes available.
Weak recommendation		
High-quality evidence	Evidence from one or more well-designed nonrandomized diagnostic accuracy studies (ie, observational ‒ cross-sectional or cohort) or systematic reviews/meta-analyses of such observational studies (with no concern about internal validity or external generalizability of the results)	The degree to which the diagnostic test is seriously considered may differ depending on circumstances or patients’ or societal values
Moderate-quality evidence	Evidence from nonrandomized diagnostic accuracy studies (cross-sectional or cohort), with one or more possible limitations causing minor concern about internal validity or external generalizability of the results	The degree to which the diagnostic test is seriously considered may differ depending on individual patients’/practice circumstances or patients’ or societal values
Low-quality evidence	Evidence from nonrandomized diagnostic accuracy studies with one or more important limitations causing serious concern about internal validity or external generalizability of the results	Alternative options may be equally reasonable.
Insufficient	Evidence may be of such poor quality, conflicting, lacking (i.e., studies not done), or not externally generalizable to the target clinical population such that the estimate of the true effect of the test is uncertain and does not permit a reasonable conclusion to be made	Insufficient evidence exists to recommend for or against routinely offering the diagnostic test.

## Results

### Audiologic diagnosis

**Figure 2:**
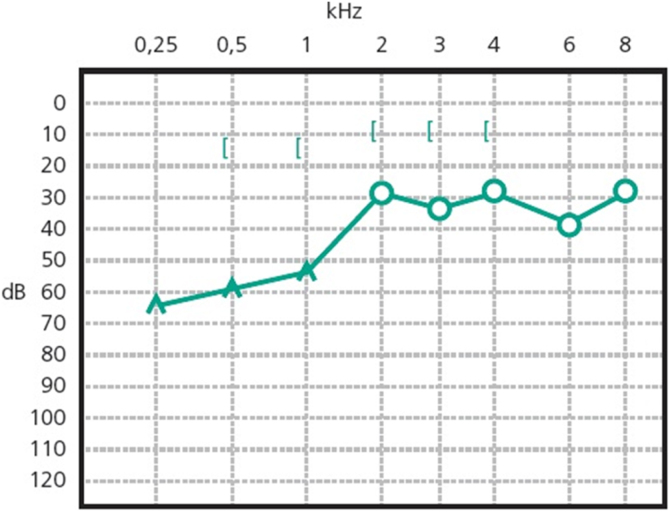
Conductive hearing loss in the left ear. Early stage of otosclerosis.

**Figure 3:**
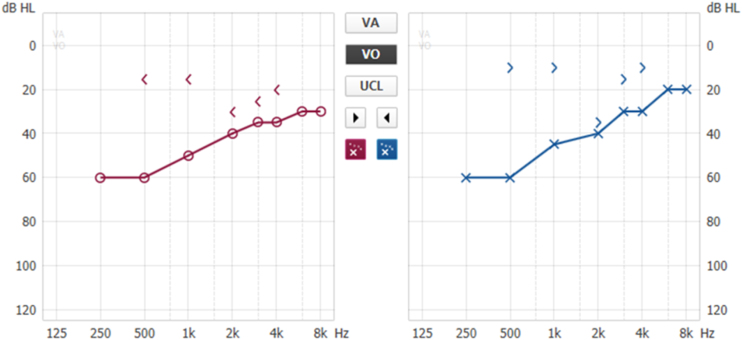
Conductive hearing loss with bilateral Carhart notch.

**Figure 4:**
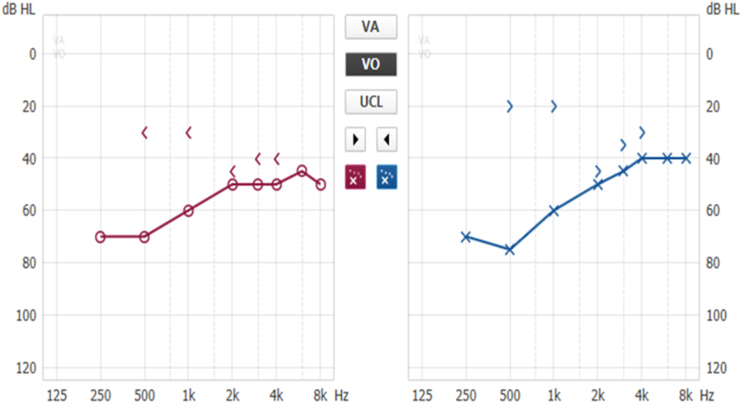
Bilateral mixed hearing loss, with bilateral Carhart notch.

Patients with otosclerosis have progressive hearing loss that is worse at low frequencies. It occurs bilaterally in 80% of patients, although unilateral involvement is often present early in the disease.^84^ Loss of bone conduction at the frequency regions around 2000 Hz (Carhart notch) has historically been considered an indicator of otosclerosis, but it is not pathognomonic of the disease^85^ (Fig. 2). Low-frequency hearing loss occurs early in the disease^86^ (Fig. 3). The progression of otosclerosis should be monitored by an audiogram because it directly correlates to hearing loss. As the stapes footplate becomes fixed to the oval window, the conductive loss worsens (increases the ABG) and begins to involve all frequencies.^86^ Occasionally, the course of otosclerosis can deviate from the classic presentation, especially in the retrofenestral subtypes of the disease when mixed hearing loss (Fig. 4) or only SNHL might occur.^87^ On immittance testing, the tympanogram demonstrates some flattening, with a type As or Ar curve, while the stapedial reflex is absent.

Although evaluation can be complemented by other tests, such as otoacoustic emissions and Auditory Brainstem Response (ABR), audiometry is mainly used for diagnosis and follow-up of otosclerosis. Otoacoustic emissions and ABR results are compatible with pure-tone audiometry, that is, if hearing thresholds are greater than or equal to 60 dB, the main waves (I, III, and V) can be found. However, the ABG can lengthen the latency of these waves, demonstrating a change in conduction.

### Impact of imaging on evaluation and treatment of otosclerosis

Radiographic findings for otosclerosis were described more than 50 years ago. Diagnosis of the disease is based on history, physical examination, and characteristic audiometric findings.^88^ Imaging is useful in the evaluation of patients before primary stapes surgery, during revision surgery, and before Cochlear Implant (CI) surgery.^19^, ^89^

Temporal bone High-Resolution Computed Tomography (HRCT) without contrast is the imaging modality to assess the otic capsules, bony labyrinth, ossicular chain, round and oval windows, and facial nerve, in addition to demonstrating the relationship of vascular structures in the posterior fossa.^90^, ^91^ Axial and coronal HRCT has been the modality of choice for otosclerosis, with sensitivity ranging from 34% to 91%.^92^ One study demonstrated sensitivity higher than 90% in most cases and the ability to describe lesions in the submillimetric scale.^88^

**Figure 5:**
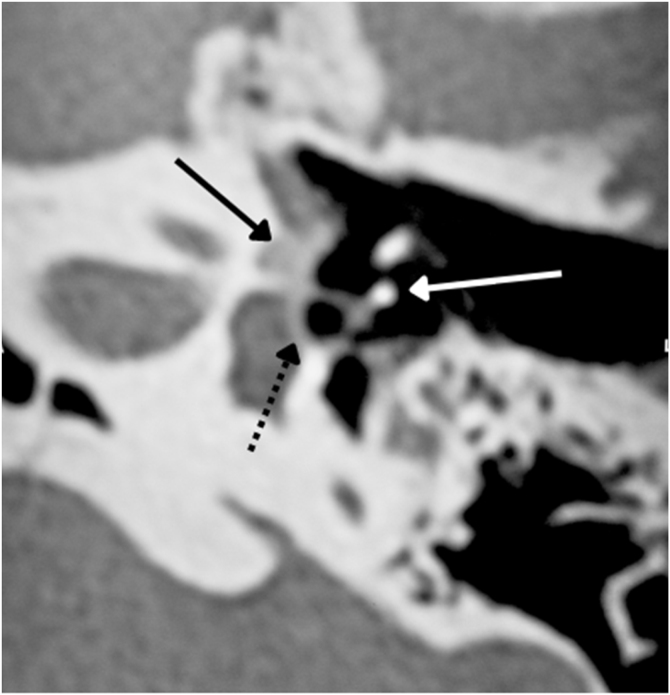
Fenestral otosclerosis. Black continuous arrow ‒ fissula ante fenestram ‒ hypodense foci of bone. Black discontinuous arrow – oval window. White arrow – stapes.

The physiologic hallmark of fenestral otosclerosis is temporal bone remodeling that occurs mainly in the area of the oval window, specifically in its anterior part, the *fissula ante fenestram*, which is a groove between the oval window and the cochleariform process. During the active (otospongiotic) stage of the disease, hypodense *foci* of bone can be identified in this area.^87^ These *foci* will be replaced later by sclerotic bone in the nonactive (otosclerotic) stage, which may progressively involve the stapes footplate resulting in its thickening and fixation (Fig. 5). This stage of the disease is manifested by progressive conductive hearing loss.^88^

**Figure 6:**
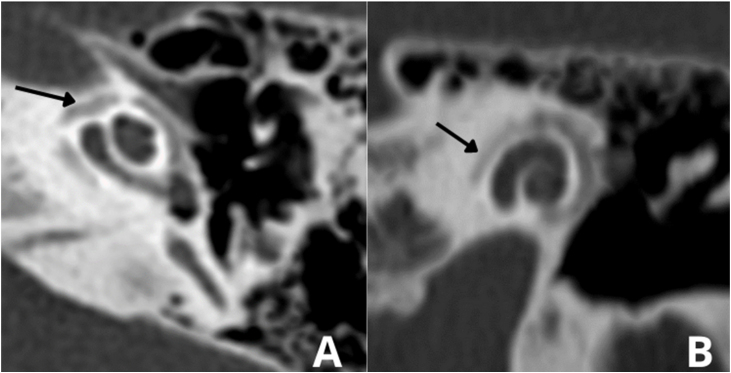
(A) Arrow indicates otospongiosis in the area of the oval window. (B) Double ring/halo sign around the cochlea showing otospongiotic stage with probable sensorineural hearing loss.

**Figure 7:**
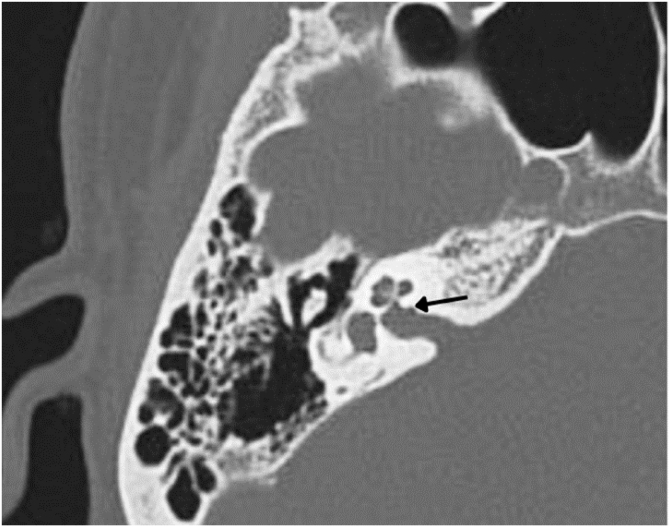
Area of demineralization of the internal auditory canal.

In 1%–10% of cases, a retrofenestral subtype of the disease occurs with the disease involving the otic capsule (Fig. 6), which can demineralize, leading to “far-advanced otosclerosis”, which has been defined by House and Sheehy^93^ as hearing loss secondary to otosclerosis with an air conduction pure-tone average of 85 dB or greater and no measurable bone conduction.^88^, ^93^ Demineralization of adjacent areas of the IAC, known as the “nipple sign” (Fig. 7), is also characteristic of retrofenestral otosclerosis.

**Figure 8:**
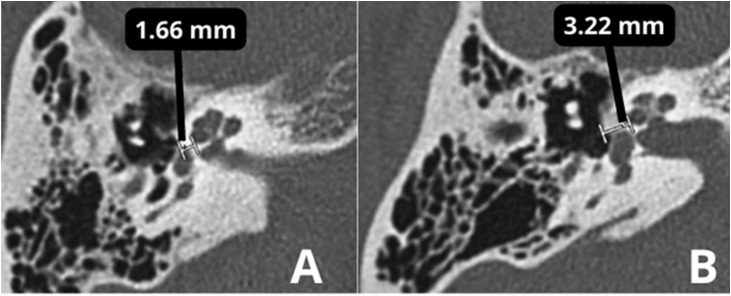
(A) Thickness in the area of the oval window is 1.64 mm (normal). (B) Thickness of 3.32 mm compatible with otosclerosis.

**Figure 9:**
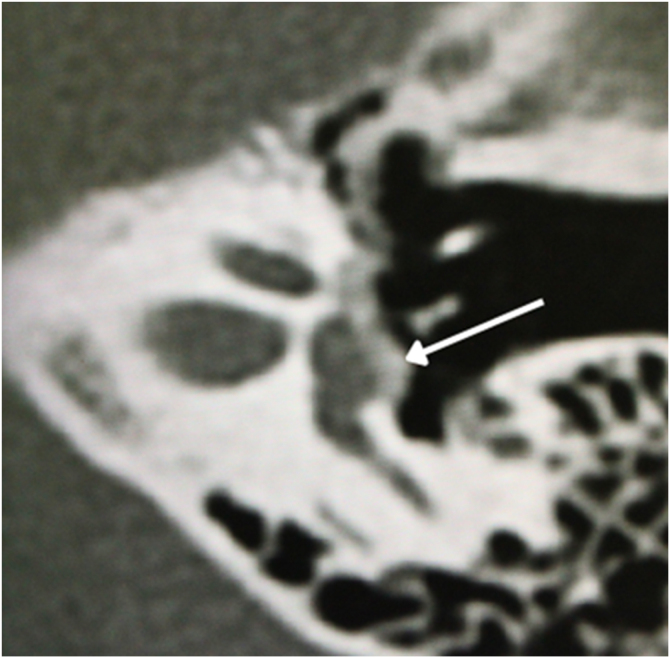
Footplate involvement by hypodense foci bone.

In the otosclerotic stage, Sanghan et al.^94^ showed that otic capsule thickness of >2.3 mm on the axial slice at the level of the cochleariform process (Fig. 8) has 68.3% sensitivity, 98.1% specificity, 97.3% positive predictive value, and 76.3% negative predictive value for differentiating patients with otosclerosis from individuals with normal hearing. Another HRCT-based modality is the densitometry measurements of the *fissula ante fenestram* area, which provide quantitative assessment of the disease and higher sensitivity.^92^ Kutlar et al.^88^ found significantly lower density in active otosclerosis than in control ears. In practice, quantitative measurements are not usually provided, despite the radiologic classifications, but rather qualitative measurements that also exhibit density lower than that of the normal otic capsule (hypodense), which may involve the entire footplate (Fig. 9) or just the anterior edge.

**Figure 10:**
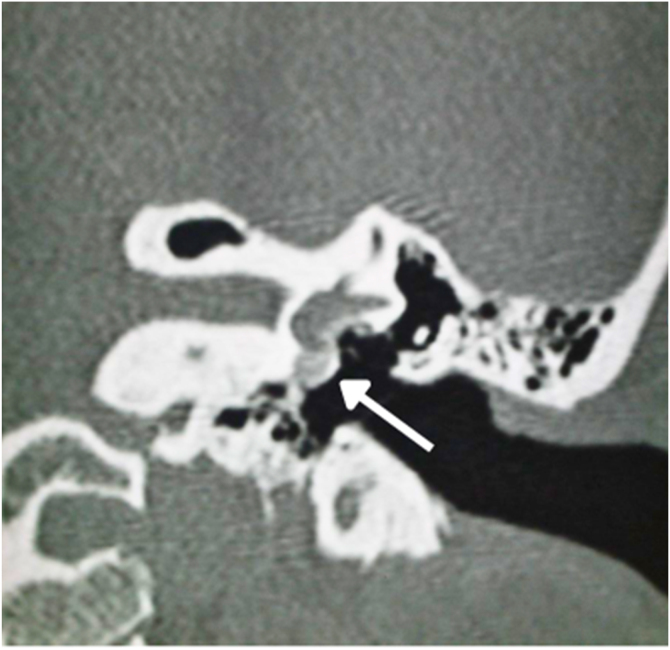
Arrow indicates otospongiosis in the area of the round window.

In stapedotomy surgery, HRCT becomes essential to assess the oval window area and its thickness, as well as the involvement of the cochlea (Fig. 5). The round window can also be partially obliterated in some cases by an otospongiotic bone block (Fig. 10), which may be a contraindication to surgery.^95^

Several classification systems have been developed for otosclerosis based on surgical and histologic findings. However, none of them are widely accepted. Multiple CT-based radiographic classification systems have been developed to describe the location and stage of otosclerosis and often the relationship between the disease radiographic stage and audiometric performance.^88^, ^96^ Rotteveel et al.^96^ demonstrated a classification system based on the histologic subdivision of otosclerosis into fenestral and retrofenestral subtypes (Table 5). An additional classification system developed by Symons and Fanning demonstrated some variation (Table 6).^97^

**Table 5:** Rotteveel classification.

CT Grading	*Foci* location
Type 1	Fenestral only (thickened footplate and/or narrowed or enlarged windows)
Type 2	Retrofenestral disease (with or without fenestral involvement)
	Double ring effect (grade 2a)
	Narrowed basal turn (grade 2b)
	Double ring effect and narrowed basal turn (grade 2c)
Type 3	Severe retrofenestral involvement (unrecognizable otic capsule), with or without fenestral involvement

**Table 6:** Symons/Fanning classification.

CT Grading	*Foci* location
Grade 1	Solely fenestral
Grade 2	Patchy cochlear disease (with or without fenestral involvement)
	To basal turn (grade 2a)
	To middle turn (grade 2b)
	Around the lateral aspects of the basal, middle and apical turns (grade 2c)
Grade 3	Diffuse confluent cochlear involvement (with or without fenestral involvement)

Classification systems may seem redundant for most cases of otosclerosis, but they are of substantial benefit in cases of retrofenestral (cochlear) otosclerosis and far advanced otosclerosis. In these cases, when patients become potential CI candidates, the choice of electrode may be influenced based on the extent of cochlear lesions in order to avoid postoperative facial nerve stimulation.^97^

Certain clinical situations may lead the clinician to suspect a diagnosis other than otosclerosis, requiring temporal bone HRCT as an additional basis for verification of the underlying diagnoses (Box 3).^87^, ^92^, ^97^, ^98^

**Box 3:** Suspected clinical conditions to indicate computed tomography.

Mixed hearing loss or significant bilateral hearing loss (in these cases, the value of audiometry may be limited because of masking, which is often not adequate)
Sensorineural hearing loss
Children with mixed hearing loss, specifically boys (to rule out X-linked mixed deafness)
Patients with facial deformity or malformation
Fluctuating hearing
History of head trauma
History of recurrent ear infections or middle/external ear surgery
Patients with associated vestibular complaints
Other causes of conductive hearing loss related to the ossicular chain

**Figure 11:**
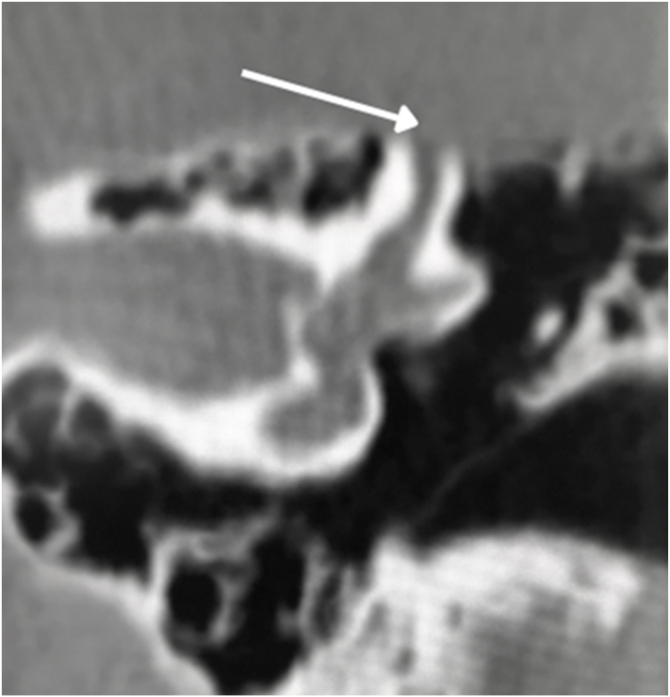
Superior semicircular canal dehiscence.

HRCT can identify other causes of conductive or mixed hearing loss, such as ossicular chain discontinuity/fixation (possibly secondary to middle ear disease), tympanosclerosis, round window obliteration, and congenital cholesteatoma.^87^, ^92^ Alternately, imaging can demonstrate different temporal bone disorders that present with conductive and mixed hearing loss, such as superior semicircular canal dehiscence (Fig. 11), osteogenesis imperfecta, Paget’s disease, fibrous dysplasia, and syphilis, as well as other rare conditions that may cause conductive hearing loss, such as granulomatous, infectious, neoplastic, and other immunologic disorders that might affect the temporal bone.^99^ Most of these conditions can be at least suspected on HRCT. Therefore, preoperative HRCT is recommended prior to surgery, being less important in patients undergoing a successful contralateral stapedectomy or stapedotomy.

**Figure 12:**
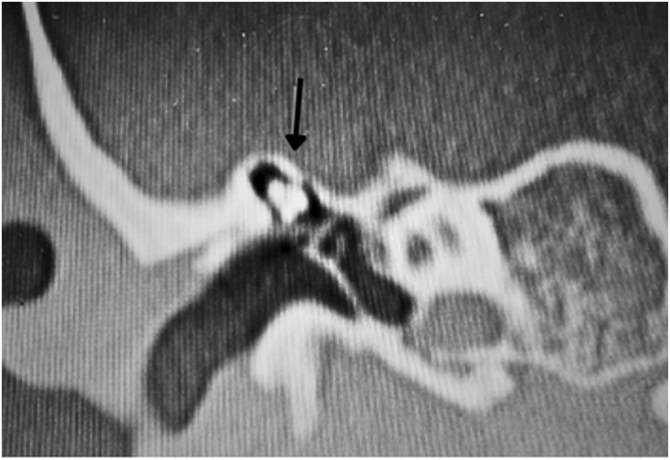
Arrow indicates malformed ossicular chain with fixation of the malleus head and incus body.

Malleus ankylosis (Fig. 12) shows an ABG in audiometry in addition to absent stapedial reflexes, and these findings are the same as those of otosclerosis. HRCT will be a particularly important test to differentiate between these findings.

**Figure 13:**
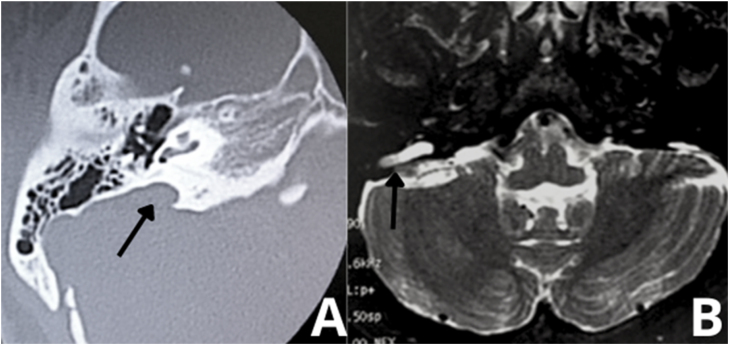
Enlargement of vestibular aqueduct (black arrows). (A) Right ear. Axial scan. Temporal bone high-resolution computed tomography. (B) MRI ‒ T2-weighted sequence of the same patient.

Preoperative imaging can also be used to avoid intraoperative complications, such as in some inner ear malformations that include enlarged vestibular aqueduct (Fig. 13) or X-linked mixed deafness, with closure defects in the fundus of the IAC. These radiographic findings lead to a significant risk of intraoperative “gusher” during stapedotomy and subsequent SNHL. Obliterated round window and ossicular fixation can lead to poor results after otosclerosis surgery if not identified before or during surgery.^100^ Assessing the location of the tympanic segment of the facial nerve is another benefit that can be derived from preoperative HRCT, which can demonstrate a dehiscent or overhanging facial nerve prolapsed into the tympanic cavity that is obstructing visualization of the oval window.^100^

The parameters that the surgeon should observe on preoperative CT in patients with suspected otosclerosis are described in Box 4.

**Box 4:** Parameters to be evaluated on temporal bone computed tomography scans in patients with suspected or diagnosed otosclerosis for stapes surgery planning.

*Fissula ante fenestram*
Thickening of the tympanic membrane to the stapes footplate
Position of the tympanic portion of the facial nerve
Otosclerotic focus in the round window
Superior semicircular canal dehiscence
Enlarged vestibular aqueduct
Signs of ossicular chain discontinuity

#### Imaging in advanced otosclerosis and cochlear implant

**Figure 14:**
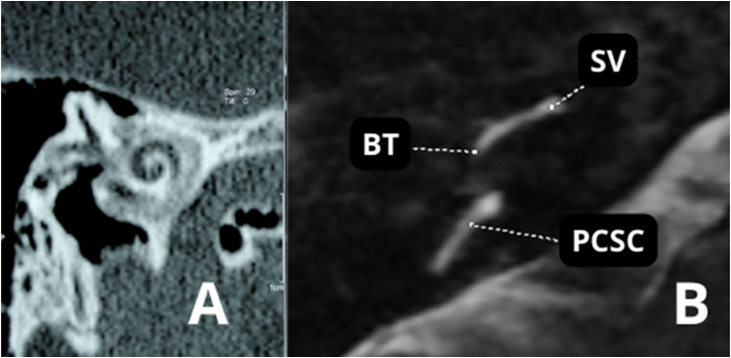
(A) Advanced otosclerosis with double ring/halo sign. (B) MRI ‒ T2-weighted sequence of the same patient showing stenosis of the scala tympani in the basal turn. BT, Basal Turn; SV, Scala Vestibuli; PCSC, Posterior Semicircular Canal.

**Figure 15:**
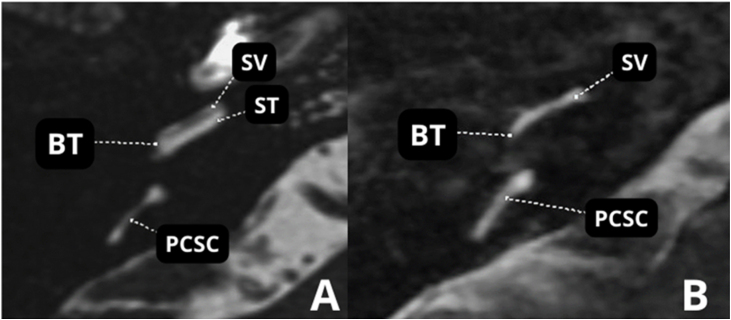
(A) MRI ‒ T2-weighted sequence A. Normal. (B) Advanced otosclerosis with showing stenosis of the scala tympani in the basal turn. BT, Basal Turn; SV, Scala Vestibuli; ST, Scala Tympani; PCSC, Posterior Semicircular Canal.

Temporal bone CT scans in patients with otosclerosis who will need a CI most often show significant changes in the otic capsule and round window. It is often impossible to detect the lumen of the scala tympani (Figs. 14A and B) secondary to labyrinthitis ossificans. Therefore, MRI in these cases is essential to detect a visible space in the scala tympani (Fig. 15A). Partial stenosis (Fig. 15B) of the Scala tympani may occur, characterized by ossification/calcification on CT and low signal on MRI, which may result from fibrosis and/or ossification in the basal turn of the cochlea.

**Figure 16:**
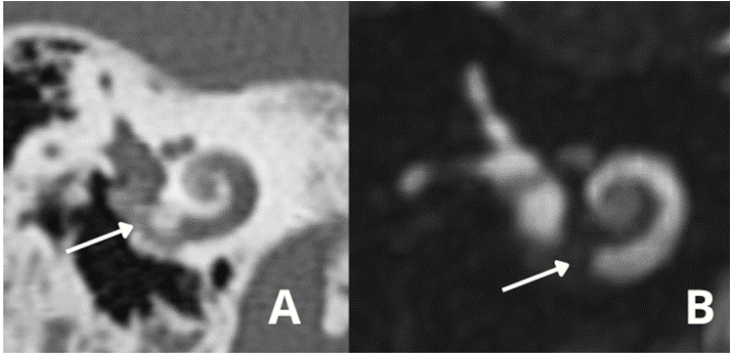
(A) Obliteration of the round window and part of basal turn.A ‒ Temporal bone high-resolution computed tomography; (B) MRI ‒ T2-weighted sequence.

Another image that should be observed is calcification of the round window, which is the preferred entry route for insertion of the CI electrode bundle (Fig. 16), especially in cases where there is the possibility of preserving hearing.^101^

Therefore, mastoid CT and MRI complement each other in CI cases, and it is important to request both tests to improve diagnosis and assess possible difficulties during surgery.^101^

MRI is not indicated for the imaging diagnosis of otospongiosis, but it may demonstrate signal alteration (hyperintensity on T2-weighted images with gadolinium enhancement) in some cases, which denotes disease activity.

#### Recommendations (Box 5)

**Box 5:** Otosclerosis diagnosis recommendations.

Patients with conductive hearing loss, with Carhart notch on the audiogram, absence of stapedial reflex, type Ar tympanogram, family history of otosclerosis, and successful stapes surgery in one of the ears gain little benefit from imaging (Moderate recommendation – Low-quality evidence).
Mastoid HRCT is the imaging modality of choice for patients with a clinical indication for stapes surgery (Strong recommendation – High-quality evidence).
MRI is weakly recommended in patients with otosclerosis and conductive hearing loss (Strong recommendation – Moderate-quality evidence).
In patients with an indication for CI, temporal bone MRI should be performed to evaluate the patency of the cochlea (Strong recommendation – High-quality evidence).

### Vestibular symptoms in patients with otosclerosis

Vertigo in patients with otosclerosis has been well known for more than 50 years. Cawthorne^102^ observed that 24% of patients with otosclerosis had episodes of dizziness. However, the pathophysiologic mechanism by which otosclerosis causes vertigo remains unclear. Three main mechanisms have been proposed: 1) Otoconia detachment, especially from the utricle, invading the endolymphatic space of the posterior semicircular canal; 2) Otosclerotic *foci* involving the vestibular system with or without neuronal degeneration; and 3) Otosclerotic *foci* involving the endolymphatic duct and sac, resulting in dysfunction of the endolymphatic resorptive system and subsequent formation of EH.^103^

Yoon et al.^104^ studied 128 temporal bones with otosclerosis, of which 10 (8%) had severe EH. Igarashi et al.^105^ studied 10 temporal bones with otosclerosis, of which 8 showed utricular distension. Vertigo has been reported to occur when saccular hydrops is large enough to fill the vestibule. Second, patients with otosclerosis may develop Benign Paroxysmal Positional Vertigo (BPPV). A histopathologic study of temporal bones showed absence of otoconia in the otolith macula close to the otosclerotic focus.^106^ Otoconia detachment invading the endolymphatic space can cause vertigo, evidenced by basophilic deposits in the posterior semicircular canal in otosclerosis.^107^

Third, in patients with otosclerosis, vertigo may be caused by damage related to the presence of *foci*. Ghorayeb and Linthicum^103^ reported that at least 1 focus of otosclerosis was in contact with the utriculoampullar branch of the vestibular nerve in temporal bones of patients with otosclerosis. Therefore, degeneration of the vestibular organ and/or neural pathway would play a different role in inducing vertigo in patients with otosclerosis.

#### Vestibular symptoms and otosclerosis surgery

Stapes surgery for otosclerosis can result in vestibular symptoms in approximately 70% of patients during the first postoperative week. Late vertigo as a complication of stapes surgery is relatively rare (5%–8% of cases).^108^ Persistent vertigo associated with a poor audiologic outcome is one of the indications for revision stapes surgery for otosclerosis. The revision surgery rate ranges from 2.5% to 13.2%.^109^

Prostheses up to 0.4‒0.6 mm in diameter can be safely applied during stapedotomy at a depth of up to 0.5 mm within the vestibule. The safest location for stapes footplate fenestration during stapedotomy is the center of the footplate. The shortest distance from the center of the stapes footplate to the utricle and saccule is 1.2 and 1.4 mm, respectively. The shortest distance to the cochlear duct was observed at the inferior edge of the stapes footplate (0.2 mm). The cochlear duct is always located below the inferior edge of the stapes footplate. Therefore, the risk of persistent vestibular damage during a correctly performed stapedotomy in the center of the footplate is virtually nonexistent.^110^

Early vertigo is usually caused by damage to the inner ear during surgery. It is mainly caused by contact between the utricle (which is located very close to the oval window) and surgical instruments or the prosthesis or as a result of perilymph aspiration. In these cases, treatment includes bed rest and adequate pharmacotherapy.

Vertigo that persists for months after surgery may have been caused by an excessively long stapes prosthesis, which extends too far into the vestibule and compresses the utriculosaccular structure. Persistent vertigo may also be caused by a perilymphatic fistula in the oval window. Therefore, choosing the appropriate prosthesis length for insertion into the vestibule is extremely important.^111^

Persistent vertigo as an indication for revision surgery in otosclerosis accounts for 2.9% of cases. Incorrect prosthesis length in primary surgery affects 5.8% of all patients undergoing revision surgery. Persistent late vertigo may result from bone fragments left in the inner ear during primary surgery, directly compressing the saccule. Other causes of late vertigo include blood penetration into the labyrinth, acute postoperative labyrinthitis, incorrect prosthesis position, and adhesions and scarring around the prosthesis.^112^

In a study comparing the occurrence of vertigo after stapedectomy vs. stapedotomy, Sakamoto et al. showed that postoperative vertigo duration was 1.0 ± 2.0 days after stapedotomy and 3.3 ± 4.0 days after stapedectomy, with a significant difference between them (*p* = 0.003). Therefore, the duration of postoperative vertigo is shorter in patients undergoing stapedotomy.^113^

#### Preoperative vestibular assessment and postoperative prognosis

Two tests have been proposed for preoperative and postoperative vestibular assessment in stapedectomy/stapedotomy: video Head Impulse Test (vHIT) and Vestibular Evoked Myogenic Potentials (VEMP). These tests also aim to detect other disorders of the inner ear that may have repercussions on surgical indication, such as Ménière’s disease.

Catalano et al.^114^ published the preliminary findings of a study investigating the role of vHIT in the evaluation of otosclerosis. There was no difference between preoperative and postoperative vHIT gains. They suggested that semicircular canal function is not modified by otosclerosis itself and does not change after stapes surgery.

However, Satar et al.^115^ investigated the effects of otosclerosis and stapedotomy on vHIT and concluded that otosclerosis and stapedotomy may affect the functions of the semicircular canals evaluated by vHIT. The lowest gain was obtained from operated ears, followed by unoperated and control ears, respectively. In terms of incidence of covert saccade, operated and unoperated ears differed significantly from control ears for lateral and posterior semicircular canals. Therefore, the results are still conflicting regarding the role of vHIT in the evaluation of patients with otosclerosis.

In VEMP testing, airway stimulation allows evoking myogenic potentials to be recorded in the contracted neck muscles, called cervical VEMP (cVEMP), and in extraocular muscles, called ocular VEMP (oVEMP). The battery of tests has been recently expanded to assess dynamic otolith function. Manzari et al.^116^ proposed that cVEMP represents predominantly saccular function and oVEMP primarily reflects utricular function, although the relative contribution of utricular vs saccular afferents to VEMP is still hotly debated.^117^ Stimuli transmitted through the middle ear conduction system have failed to elicit cVEMPs in ears with conductive hearing loss, i.e., chronic otitis media or otosclerosis.^118^ To overcome the attenuation of stimulation caused by middle ear disease, bone conduction stimulation has been used to induce cVEMPs. However, the stimuli are not consistent, and the method limits their clinical use.^119^ In the early stage, localized fibrous fixation of the footplate may not hinder sound transmission. As the disease progresses to an advanced stage, either diffuse fixation of the footplate or ankylosis of the entire ligament can lead to an absence of cVEMPs even with the use of bone conduction stimulation.

Therefore, the use of electrophysiologic tests for preoperative and postoperative evaluation of patients who will undergo stapes surgery is still controversial.

Regarding prognostic evaluation, studies indicate that previous surgery in the contralateral ear is the main poor prognostic factor for persistent spontaneous nystagmus and prolonged vertigo after stapedotomy in the opposite ear.^120^

#### Otosclerosis surgery and vestibular disorders

Otosclerosis surgery is commonly indicated in patients with other vestibular disorders, such as Ménière’s disease. According to the study by Shiosansi et al.,^121^ stapes surgery provides excellent outcomes for most patients with Ménière’s disease, even though fluctuating hearing and progressive cochlear degeneration may occur. Thus, concomitant Ménière’s disease would not be a contraindication. The study included 15 patients with a clinical diagnosis of Ménière’s disease, being only indicated after Ménière’s disease was considered clinically stable for at least 6 months without fluctuating hearing. However, as the sample was small, this indication should be done with caution.^122^

Likewise, according to Shiosansi et al.,^90^ the coexistence of otosclerosis with superior semicircular canal dehiscence syndrome would not be a contraindication to surgery. However, residual conductive hearing loss can be expected after surgical treatment, while the onset of new symptoms of the syndrome after otosclerosis surgery is rare.^123^

Therefore, concomitant vestibular disorders, such as Ménière’s disease and superior semicircular canal dehiscence, may not be a contraindication to surgery, but patients should be informed of the possible different audiologic outcomes in these scenarios.

#### Recommendations (Box 6)

**Box 6:** Recommendations ‒ Vestibular symptoms in patients with otosclerosis.

Stapedotomy is associated with a lower incidence of vertigo postoperatively compared with stapedectomy (Low-quality evidence).
It is recommended that the fenestra during stapedotomy be made in the central part of the footplate (Insufficient evidence).
Previous surgery for otosclerosis in the contralateral ear increases the likelihood of postoperative vertigo after surgery in the opposite ear (Low-quality evidence).
Ménière’s disease or superior semicircular canal dehiscence are contraindications to stapedotomy (Insufficient evidence). If indicated in Ménière’s disease, it is recommended that the disease be clinically stable for at least 6 months without fluctuating hearing (Insufficient evidence).

## Discussion ‒ treatment

### Stapes surgery

#### History

Stapes fixation was first described as a cause of hearing loss by Antonio Maria Valsalva in 1704 after dissection of a deaf patient. In 1841, Toynbee dissected 1659 temporal bones and found stapes fixation in 39 of them, concluding that “osseous ankylosis of the stapes to the fenestra ovalis was one of the common causes of deafness”. However, chronic inflammatory processes in the middle ear were believed to be responsible for secondary ankylosis of the stapes. In 1893, Adam Politzer described the histologic findings of 16 cases of stapes fixation, which indicated that the deafness was due to a primary disorder of the labyrinthine capsule. He referred to this disease as otosclerosis.^124^

In 1842, Prospere Ménière reported the case of a patient who temporarily improved his own hearing by tapping the stapes directly with a small gold rod. Johannes Kessel was the first to describe stapes surgery in 1876. He believed that the hearing loss associated with otosclerosis was caused by increased pressure on the inner ear fluids. Based on experimental research in pigeons, he performed stapes mobilization and removal in humans. He would first separate the incus from the stapes and then attempt to mobilize the stapes by applying pressure to its head in various directions. When this was not successful, he would remove the stapes. Kessel reported some improvement in hearing and no serious complications. However, his findings differed from other physicians. In many cases, the hearing improvement only lasted for days or weeks and with the risk of labyrinthitis and meningitis.^125^ In 1899, Kessel was harshly criticized by some of the leading surgeons of the time, such as Politzer, Siebenmann, and Moure, at the 6th International Otology Congress in London. During this meeting, stapes surgery was declared “useless, often mutilating, and dangerous”. In 1900, Johannes Kessel was publicly censured for unscrupulousness.^124^

Because stapes surgery was considered too dangerous, surgeons started using “third-window” fenestration techniques. At the end of the 19th century, Passov and Floderus proposed the idea of a fenestration on the promontory or vestibular labyrinth, but it did not become fully established until 1913, when Jenkins described fenestration of the lateral semicircular canal. Several surgeons developed fenestration techniques – Holmgren, Sourdille, and Julius Lempert. Lempert’s contribution was to simplify the fenestration technique that was previously performed in three stages to only one stage. The single-stage endaural approach to fenestration was a significant improvement of Sordille’s three-stage approach.^12^, ^126^ The hearing results were consistent: more than 50% of patients reported hearing gains of 20–25 dB. Lempert’s technique became the main technique for otosclerosis in the 1930s and 1940s.^124^, ^127^

Samuel Rosen was the first to describe stapes mobilization in the mid-twentieth century. Rosen used Lempert’s technique; however, before performing the fenestration, he would check for the mobility of the stapes to ensure it was fixed. In 1952, almost by accident, Rosen developed the operation that would make him famous. During a routine procedure, Rosen accidentally mobilized the stapes while tapping on it to check for fixation. The patient, who was awake during the procedure, started noticing sound coming from the operating room next door.^128^ Rosen’s procedure was performed under local anesthesia via a transcanal approach. Patients had immediate results on the operating room table, and the recovery period was short. The surgery was relatively simple when compared with Lempert’s fenestration operation and was easy to teach. The shortcoming of the mobilization procedure was that many patients would refixate shortly after the operation. Rosen would often have to perform revision surgery. After more than half a century, stapes surgery was finally reestablished.^127^, ^129^

John Shea, by reading the literature on stapes surgery from the end of the 19th century, realized the significance of the procedure described by Frederick Jack about a patient who maintained good hearing for 10 years after stapes surgery, and that it must be possible to remove and replace a stapes fixed by a prosthesis. In a female patient with otosclerosis, after removing the stapes and sealing the oval window with a subcutaneous tissue, Shea used a Teflon prosthesis to replace the stapes for the first time on May 1, 1956, with complete success.^126^ At the time of Shea’s discovery, complete stapes removal was still considered too dangerous and was forbidden. Within a decade, Shea’s stapedectomy procedure became the standard operation for the treatment of otosclerosis. In the 1960s, thousands of patients with impaired hearing due to otosclerosis were treated with great success. In 1960, Schuknecht developed a steel-wire prosthesis to address both the need to seal the vestibule and to reconstruct the ossicular chain.^130^ As the stapedectomy procedure evolved, several methods to remove just a part of the footplate emerged. The procedure was modified so that only a small fenestra was created.

#### Indications and contraindications to stapes surgery

##### Indications for stapes surgery (Box 7)

**Box 7:** Classical conditions for indication of stapes surgery.

Diagnosis of otosclerosis
Conductive hearing loss with a mean tonal threshold ≥25 dB at 250 Hz, 500 Hz, 1000 Hz, and 2000 Hz and
ABG ≥ 20 dB^6^, ^131^, ^132^
Negative Rinne’s test at 512 Hz^133^ in the affected ear.

##### Contraindications (Box 8)

**Box 8:** Contraindications to stapes surgery.

Ear with evidence of otosclerosis, but contralateral side with profound deafness
Active infection of the outer and/or middle ear
Tympanic membrane perforation
Active Ménière's disease
Unfavorable clinical condition
Occupational or recreational condition requiring intact vestibular function
Persistent stapedial artery

#### Special situations

##### Age

Stapes surgery is a safe treatment option in children with otosclerosis that has good hearing outcomes.^134^ Although studies have not established a minimum age for the procedure, Vincent et al.,^134^, ^135^ in addition to showing their results, conducted a literature review of 14 studies that corroborated the safety and hearing gains of stapes surgery in children aged ≥5 years.

##### Chefs and sommeliers

Surgery should be reconsidered in certain professions. Chefs and/or sommeliers (of wine or other beverages) should be alerted to possible permanent taste disorders (after 1 year of the procedure) after the surgery.^136^ Other methods of auditory rehabilitation should be considered, such as the use of a Personal Sound Amplification Product (PSAP). If the patient still wants the surgery, a specific term informing about the risk of loss of work function after the procedure should be elaborated.

##### Aviation

Thiringer & Arriaga^138^ examined 16 US Air Force aircrew members who had undergone stapedectomy and returned to flight duty after a series of otologic tests to assess fitness to return to work. All prostheses were variations of the piston, and oval window seal was documented in 4 patients, including vein, fascia, fat, and Gelfoam. None of the 16 aircrew members reported any symptoms related to the stapedotomy procedure during flight. Katzav et al.^139^ reported 9 stapedotomy procedures in 6 high-performance airline pilots in the Israeli air force who returned to flight duty shortly after 3 months after surgery, without any vestibular symptoms. There is no evidence in the current literature that supports the contraindication of stapedotomy/stapedectomy in this setting. If surgery is chosen, all possible complications (such as permanent damage to the vestibular system) must be detailed to the patient, and the patient must be informed of the possibility of loss of work function.

In Brazil, military pilots are not considered fit to work after undergoing stapes surgery, according to the last Technical Instruction of the Health Inspections – Air Force Command of 2016.^140^ In the civil sphere, the 2021 position from the National Civil Aviation Agency does not specify stapedotomy/stapedectomy as a limitation for the qualification of first- and second-class medical certificates (the latter includes flight attendants), but clearly specifies that those with permanent labyrinthine disorders cannot be certified.^141^

##### Diving

The professional or recreational practice of scuba diving may represent an increased risk of perilymphatic fistula and prosthesis displacement by barometric stress. There is no strong evidence in the literature to corroborate this hypothesis. Published studies did not show an increase in the risk of labyrinth and cochlea injuries with the practice of scuba diving.^142^, ^143^ Harrill et al.^137^ sent a questionnaire on postoperative management of patients undergoing stapes surgery to members of the American Society of Otology and Neurotology. They found that 54.3% of surgeons who performed a stapedectomy or stapedotomy recommended permanent diving restriction.

House et al.^142^ identified 22 patients who returned to diving after undergoing a stapedectomy; 4 of them presented otologic symptoms, including otalgia (3), tinnitus (1), and transient vertigo (1). One patient developed sudden SNHL and vertigo 3 months after scuba diving. A perilymphatic fistula was found at examination and successfully repaired. The perilymphatic fistula was not believed to be related to diving due to the delay between symptom onsets. This patient continued to dive without problems after repair of the perilymphatic fistula. The authors concluded that there is no increased risk of barotrauma with diving after stapedectomy provided that adequate tube function has been established. Despite these reports tolerating high-performance diving and flying after stapes surgery, it is important for surgeons to address the potential risks of barotrauma with any patient undergoing stapes surgery. Furthermore, sealing the oval window with a tissue graft may provide an extra measure of safety for these patients at high risk of barotrauma.

##### Persistent stapedial artery

In a literature review and retrospective study conducted by Goderie et al.^144^ and Sioshansi et al.,^145^ respectively, there were no postoperative complications in patients undergoing stapedotomy with manipulation of the Persistent Stapedial Artery (PSA). When present (in the postembryonic period), the stapedial artery gives rise to the middle meningeal artery and may be involved in the supply of blood to the facial nerve; its course within the middle ear is closely related to this nerve.^145^ Despite the promising results shown in these studies, PSA management can lead to significant intraoperative bleeding (which makes stapedotomy more challenging) and to complications related to facial nerve and central nervous system ischemia.^144^, ^145^ In these cases, the authors recommend interrupting the procedure.

#### Recommendations (Box 9)

**Box 9:** Recommendations for stapes surgery in special situations.

Patients whose work function depends on accurate taste function should be informed of the risk of temporary or permanent occupational disability after surgery (Strong recommendation – Low-quality of evidence).
There is no evidence to contraindicate surgery in aircrew members. However, before recommending stapes surgery, the local legislation for each specific function should be checked to avoid the risk of occupational disability (Strong recommendation – Low-quality evidence).
There is no evidence that diving, or scuba diving increases the risk of hearing loss or perilymphatic fistula in patients undergoing stapes surgery, provided the patient’s tubal function is adequate. However, due to the poor quality of published studies, patients who engage in diving/scuba diving should be informed of possible risks (Moderate recommendation – Low-quality evidence).
Although some studies have shown the possibility of performing stapedotomy in patients with PSA, as there are other methods of auditory rehabilitation and due to the high risk of complications, stapes surgery is not indicated in these cases (Strong recommendation – Low-quality of evidence).

#### Surgical technique

Stapedotomy is currently the most accepted surgical treatment for fenestral otosclerosis with good cochlear reserve. Some surgeons prefer local anesthesia or local anesthesia with sedation to assess intraoperative auditory and vestibular response, whereas others prefer general anesthesia for the patient’s comfort. In 2008, Vital et al.^146^ compared the incidence of profound hearing loss among 160 patients undergoing stapedectomy under general anesthesia vs. 108 under local anesthesia and found a higher incidence of profound hearing loss in the general anesthesia group (1.8%) compared with the local anesthesia group (0%). A systematic review compared local vs general anesthesia in 417 procedures and found no statistical difference in postoperative ABG, worsening SNHL, or postoperative vertigo.^147^

Although any method of anesthesia may be equally acceptable in primary surgery, local anesthesia or local anesthesia with sedation has an advantage in revision surgery. If a patient experiences vertigo while the surgeon is manipulating or removing the previously placed prosthesis, this may indicate the presence of adhesions between the prosthesis and the saccule. Without patient feedback, the surgeon may continue to manipulate or remove the prosthesis, putting the patient’s hearing at risk.

An effective way of delivering topical anesthesia is using 1%–2% lidocaine hydrochloride with 1/100,000 adrenaline. Although lidocaine has a short half-life (between 1.5 and 2 h after intravenous bolus injection), it is indicated for typically being a quick procedure. The total dose administered in the various injection sites is approximately 10 mL and should not exceed 7 mg/kg.^148^ Infiltration starts in the retroauricular region to block branches of the cervical plexus (lesser occipital nerve and greater auricular nerve) and the vagus nerve (Cranial Nerve [CN] X) innervating the posterior surface of the outer ear and continues between the tragus and the helix. The needle must be advanced until contact with the bone to block the tympanic branch of the auriculotemporal nerve. Finally, the posterior part of the External Auditory Canal (EAC) is infiltrated to block branches of the facial nerve (VII CN) innervating the concha.

Total intravenous anesthesia reduced bleeding in studies with endoscopic surgery.^149^ Because total intravenous anesthesia causes less vasodilation than inhalational anesthesia, it reduces both mean arterial pressure and heart rate in patients, decreasing cardiac output and bleeding. Injectable local anesthetics are beneficial for achieving hemostasis during general anesthesia. Infiltration of 1 mL of 1% lidocaine with 1/100,000 adrenaline can be performed in the EAC laterally to the osteocartilaginous junction. As an adjuvant to obtain local vasoconstriction, cotton pledgets soaked in 1:1000 epinephrine can be used and positioned inside the EAC for approximately 5 min while the trichotomy is performed, if necessary.

The approach to stapes surgery has evolved over the years. Some surgeons prefer the classic transcanal approach, while others advocate using an endaural approach to increase exposure. More recently, endoscopic surgery has been used for stapedotomy. Those who advocate using endoscopes cite improved visualization, reduced need for scutum curettage, and decreased chorda tympani manipulation.^150^, ^151^ Proponents of traditional endaural and transcanal approaches point to limitations of the endoscopic approach such as loss of depth perception, potential for thermal injury to the chorda tympani, difficulty using the microdrill, and having to place the prosthesis with one hand. Despite these concerns, audiologic outcomes are comparable according to recent reports.^151^

Analyzing the risks of thermal injury to middle ear structures, Dundar et al.^152^ measured changes in oval window temperature during endoscopic stapedotomy in a guinea pig model. The authors concluded that using a 4 mm endoscope with a xenon light source caused the highest temperature increase, whereas the lowest temperature increase occurred with a 3 mm endoscope with a LED light source.^152^

The classic technique involves removing the superstructure of the stapes, then performing the fenestration and placing the prosthesis. In 1994, Ugo Fisch proposed reversing these steps during stapedotomy, in an attempt to minimize the risk of floating footplate, inner ear injury, and ossicular chain dislocation.^153^, ^154^ Instead of removing the superstructure of the stapes and then performing the fenestration, Fisch proposed first performing the fenestration and then replacing the prosthesis, still with both the incudostapedial joint and stapedius tendon intact. After the prosthesis is secured, the stapes and the lenticular process of the incus are separated, the stapes crura is fractured, the stapedius tendon is cut, and its superstructure is consequently removed. The inversion of surgical steps reduces the time of vestibule exposure, ensuring minimal blood entry into the vestibule and consequently reducing the need for manipulation and the chance of injury to the inner ear.^153^ An additional advantage of the so-called Fisch’s reversal steps stapedotomy is the increased stability of the ossicular chain, making it easier to place the piston in the long process of the incus.

According to Malafronte et al.,^154^ not all cases of otosclerosis benefit from the reversed technique. Fisch’s reversal steps stapedotomy is more indicated when the visible portion of the footplate is blue in all its points (known as “blue otosclerosis”), in which the footplate is strongly welded to the rim of the oval window, as the bone is healthy and elastic and quite resistant to trauma. In this case, Fisch’s original idea avoids incus and footplate complications. When the visible portion of the footplate, before removal of the stapes superstructure, is white in all or most of its points (“white otosclerosis”), Fisch’s reserved technique is not recommended because it does not prevent incus luxation/subluxation and floating footplate. In white otosclerosis, the footplate is well welded to the annular ligament by the otosclerotic focus that involves most of the footplate, which becomes white, fragile, and less resistant to trauma.^153^, ^154^

While Shea originally removed the entire footplate, more limited removal is currently preferred by most surgeons performing stapedotomy.^13^ In some cases of fixation limited to the anterior footplate, the laser stapedotomy minus prosthesis technique is used. In this technique, the anterior crus is separated from the footplate using a laser, allowing complete mobility of the posterior footplate despite anterior fixation. Although in one study the laser technique resulted in improved high-frequency hearing compared with small fenestra stapedotomy, it was associated with a higher rate of revision surgery for refixation. Furthermore, this technique can only be used in selected cases of otosclerosis limited to the anterior footplate and favorable anatomy.^155^

The creation of a small fenestra is the most used approach. When comparing stapedectomy vs stapedotomy, Fisch^156^ concluded that stapedotomy is the procedure of choice because it achieves better hearing results and is less traumatic to the inner ear than stapedectomy. Despite the universal acceptance of stapedotomy, there are conflicting opinions on how to best create the fenestra and what size the fenestra should be. To create the fenestra, some surgeons advocate using a diamond microdrill, whereas others prefer using a laser due to the lack of mechanical trauma; some even prefer using a combination of both.

Different types of lasers have been used in stapes surgery, including argon, Erbium-doped Yttrium Aluminium Garnet (Er:YAG), potassium-titanyl-phosphate (KTP), 532 nm diode, and CO_2_ laser systems. Advocates of the CO_2_ laser highlight increased energy absorption by the perilymph, which reduces the penetration of energy into the vestibule. However, the CO_2_ laser beam is invisible to the human eye and originally required a micromanipulator. Advances in optical fiber technology have led to a fiber-optic delivery system with a separate beam for CO_2_ lasers. Despite the theoretical advantages of using the Er:YAG or CO_2_ laser, based on the maximum absorption of their beams by the perilymph, a recent study by Kamalski et al.^157^ showed no difference in hearing outcomes or complications when comparing KTP, Er-YAG, and CO_2_ lasers.

Reviewing optimal fenestra diameter, fenestra sealing technique, type of prosthesis used, and technique to determine appropriate prosthesis length, a temporal bone study conducted by Wegner et al.^158^ showed that the use of 0.6 mm- and 0.8 mm-diameter pistons resulted in better hearing results compared with smaller diameter pistons. The use of a 0.6 mm piston was predicted to cause an ABG of 8–12 dB, whereas the use of a 0.4 mm piston was predicted to cause an ABG of 15–20 dB. (egner, 2016, The Effect of Piston Diameter in Stapedotomy for Otosclerosis: A Temporal Bone Model) Sennaroglu et al. (Sennaroğlu, 2001, Effect of teflon piston diameter on hearing result after stapedotomy) reported that using a 0.8 mm prosthesis over a 0.6 mm prosthesis leads to better hearing outcomes. Despite these results, clinical studies by Fisch^156^ analyzing long-term hearing outcomes with 0.4 mm vs. 0.6 mm pistons showed similar results at long-term follow-up for both diameters. However, Fisch reported that using the 0.4 mm piston is relatively easier, particularly for the reversed stapedotomy technique used by him (the piston is placed after the fenestra is created, before the superstructure is removed).

After fenestration, the surgeon must decide whether to seal the fenestra or not. Some surgeons advocate sealing the fenestra with a connective tissue or venous graft before placing the prosthesis to prevent perilymph loss, whereas others prefer to place connective tissue around the prosthesis after it was placed in the fenestra. Some surgeons do not place any soft tissue around the fenestra and instead allow blood to pool around the prosthesis in the fenestra. Although, in theory, the surgeon should use a tissue seal to try to limit perilymph loss, there is no evidence corroborating an increased incidence of SNHL or perilymphatic fistula when a tissue seal is not used.

A plethora of stapes prostheses are currently available, with some requiring manual crimping, some that dent with heat activation, and others that require no friction. Regardless of prosthesis type, it is important that minimal pressure be exerted on the long process of the incus and that the connection to the incus is tight enough to prevent vibration. To date, no type of prosthesis has proven to be clearly superior to another, and the decision depends primarily on the surgeon.

In addition to the debate over optimal piston diameter, the method for measuring the prosthesis length may also vary. Some surgeons measure from the top of the long process of the incus and subtract 0.25 mm, whereas others measure from the undersurface of the incus and add 0.25 mm. Only a few surgeons do not measure the prosthesis and use a standard-length prosthesis for all procedures. Ideally, the selected prosthesis should extend into the vestibule by only 0.25 mm to 0.5 mm,^159^ which allows sufficient distance between the piston and the underlying saccule. If the surgeon does not measure the prosthesis, it may extend too deep into the vestibule and cause vertigo and hearing loss.

#### The use of the endoscope vs. microscope

The microscope has been widely used in middle ear surgery over the decades. Binocular vision and the possibility of operating using both hands are very beneficial during surgery. However, depending on the region, the microscope has limited visualization, often requiring the performance of additional procedures to clear the surgical field, such as endaural incision and drilling of the auditory canal or the scutum, as well as frequently repositioning the surgeon and patient during the surgery.^132^, ^160^

The main advantage of endoscopic surgery for otosclerosis is the wide angle of view of the surgical field, which, in addition to reducing the need for scutum removal, provides better exposure when teaching and training new surgeons.^150^, ^161^, ^162^ The wide-angle view provided by the endoscope allows a closer and more accurate visualization of the footplate while reducing the extent of bone removal from the scutum, in addition to reducing the need for manipulation of the chorda tympani nerve.^160^, ^163^, ^164^ Although these advantages have been described by almost all authors, a precise or minimally objective method for assessing improved visibility is currently lacking, meaning that one of the main advantages of endoscopic surgery is based on the individual experience of each surgeon.^165^ It should also be noted that the endoscope reduces depth perception by only allowing a two-dimensional view, in addition to requiring the use of only one hand to operate, which may hamper the management of the procedure and possible intraoperative complications.^160^, ^164^, ^166^ The association of these factors leads to a greater learning curve, which is why surgeons who commonly use the microscope prefer to continue using it.

Another issue related to endoscopic surgery is the diameter of the endoscope. Endoscopes were initially used in Otorhinolaryngology for nasal surgeries and thereby 4 mm nasal endoscopes were more common. They were only later introduced in the field of Otology, which led to the widespread use of 3 mm endoscopes. However, no study has been able to prove the superiority of narrower endoscopes, as both hearing outcomes and complication rates were similar in patients operated on with either diameter,^167^, ^168^ despite reports of improved visibility with a smaller endoscope.

The introduction of the endoscope in the operating room did not change the surgical technique commonly used by surgeons, but rather provided an alternative access route. Therefore, it would be logical to assume that hearing outcomes remained similar to those of microscopic surgery. This assumption was confirmed by two recent systematic reviews that revealed a very similar ABG closure in all reported frequencies, with no statistically significant difference. According to Koukkoullis et al.,^169^ there would be a trend toward greater success in ABG closure with the endoscope if the study by Sproat et al.,^170^ one of the studies with the largest population and the only to use an instrument specifically designed for otologic procedures for data collection, had not been included in the systematic review.^17^ The length of experience of the surgeon should also be considered, as it may constitute an important bias. In accordance with these findings, Molinari et al.^171^ published a retrospective study in which the same surgical team was evaluated regarding to operating time for endoscopic surgery at two different time points, with a 3 year different between each assessment. The authors found that as the surgical team gained experience, the operating time for the same surgery decreased.

As for complications involving injury to the chorda tympani nerve and consequent dysgeusia as well as residual perforation, no study has found a significant difference between endoscopic and microscopic procedures.^160^, ^161^, ^168^, ^171^^,^^172^ Some studies have even associated the use of the endoscope with a lower rate of chorda tympani nerve injury due to the reduced need for scutum removal to improve visualization.^20^, ^167^ However, a direct relationship between the extent of necessary bone curettage and the occurrence of neural injury or dysgeusia in the immediate or late postoperative period cannot be established.^171^ Furthermore, a recent systematic review comparing microscopic and endoscopic stapes surgery found that these complications occurred more frequently in patients undergoing endoscopic surgery.^169^ As possible causes, the authors suggested the loss of three-dimensional (3D) view, which would facilitate the inadvertent use of sharp instruments, and the longer learning curve.

Postoperative dizziness is a very common symptom and varies greatly from patient to patient in terms of intensity and duration. Although expected and frequent, it causes significant discomfort and, when prolonged, substantially affects quality of life. Loss of 3D view and loss of depth perception have been suggested to cause increased dizziness in the postoperative period of endoscopic surgery due to inaccurate measurement of prosthesis length or window overheating due to direct incidence of endoscope light sources.^150^, ^160^ However, published meta-analyses have not been able to prove this. On the contrary, they found that dizziness outcomes were similar in groups undergoing microscopic and endoscopic surgery, and that dizziness is more related to prosthesis length and trauma at the time of fenestration than to surgical approach.^168^, ^173^, ^174^

Finally, although some studies report the advantages of using one approach over the other, there is consensus that one cannot be actually considered superior to the other. Importantly, the choice of surgical approach should be based on the surgeon’s experience, training, and availability of adequate tools for the safe performance of stapedotomy or stapedectomy.

#### Recommendations (Box 10)

**Box 10:** Recommendations for endoscopic stapes surgery.

The use of the endoscope in stapes surgery is equally as safe as the use of the microscope (Strong recommendation – High level of evidence).
The surgeon’s expertise has more impact on the surgical outcome than the chosen surgical approach (Strong recommendation – High level of evidence).
Endoscopic stapes surgery has a shorter operating time than microscopic surgery (Weak recommendation – Low level of evidence).
Endoscopic stapes surgery has comparable audiologic outcomes to microscopic stapes surgery (Strong recommendation – High level of evidence).
The use of a 3-mm endoscope is essential for performing endoscopic middle ear surgery (Weak recommendation – High level of evidence).

#### The use of laser and microdrill in stapedotomy

Fenestration techniques in stapedotomy have been modified over time with the use of microinstruments, microdrills, and eventually lasers. Conventional techniques using manual drills are widely used and surgeons are familiar with them. The footplate can be easily and safely drilled, especially if thin, by hand drills.

##### Microdrill

The microdrill used in stapedectomy has low noise intensity and low torque. A small diamond burr (usually 0.6 mm or 0.7 mm in diameter) is used, which has been shown to be safe for footplate drilling and to not cause acoustic trauma. The microdrill is safe and effective in difficult cases such as narrow footplate and facial nerve dehiscence.^175^ Drilling in otologic surgery has been suggested to have negative impacts such as trauma, vibration, and consequent hearing loss, especially at high frequencies.^176^ Kylén et al.^175^ analyzed possible factors that increase drill-generated noise levels, suggesting that the size of the burr is an important factor – smaller, diamond burrs generate less noise.

Mangham et al.^177^ reported better hearing results with the use of the microdrill compared with the hand drill. In addition to not causing cochlear damage, the microdrill has advantages such as ease of use. The perforation is performed in seconds, and the hole is round with regular margins, similar to those of the prosthesis. There is little space between the prosthesis and the hole margin, reducing the possibility of fistula and the need to fill the surrounding area of the prosthesis with tissue. Thus, there is reduced chance of granulation and scar tissue formation.^178^

Retrospective and prospective observational studies and unblinded trials did not show superiority of the microdrill over microperforators. When comparing the use of a perforator with a microdrill, Gjuric et al.^179^ found the same postoperative gain, without evidence of greater trauma to the inner ear with the use of a microdrill.

Yavuz et al.^178^ demonstrated that both the perforator and microdrill can be used without the risk of damaging the inner ear and causing footplate mobilization. The authors did not find one method to be superior to the other with regard to ABG closure and complication rates.

Canale et al.^180^ analyzed patients undergoing stapedotomy with a microdrill and found good audiologic outcomes (postoperative ABG < 10 dB) even in patients with small ABGs. A mobile footplate is more likely to be found in patients with small ABGs, which means these patients are at increased risk of floating footplate during fenestration.

One of the indications for using a microdrill is obliterative otosclerosis. Conway et al.^181^ compared the postoperative results of patients with obliterative otosclerosis undergoing microdrill fenestration vs patients without obliterative otosclerosis undergoing laser surgery. The results were similar in both groups.

Microdrill stapedotomy for footplate fenestration is an effective surgical technique. A few drawbacks of using the microdrill include the possibility of advancing into the vestibule and causing SNHL, as well as vertigo due to perilymphatic gusher. One of the limitations of endoscopic surgery is reduced depth perception, which may complicate the use of a microdrill in endoscopic surgery.^181^ Kaul et al.^182^ conducted a prospective study comparing the use of a microdrill in endoscopic vs microscopic surgery. They found no difference in hearing results. The only difference was in operating time, which was on average 10 times longer in endoscopic surgery.

##### Laser

Lasers were first used in otosclerosis surgery by Palva in 1979 for footplate fenestration; they emerged with the objective of further reducing the mechanical manipulation of the footplate and, consequently, of the inner ear.^183^ The laser allows the surgeon to perforate the footplate without directly touching it (“no touch”). In 1980, Perkins et al.^184^ described the use of argon laser in 11 patients. Multiple small holes were created in a rosette fashion, and no patient experienced SNHL.

Compared with conventional techniques, the laser minimizes the mechanical risk of trauma to the inner ear due its capacity to cut, vaporize, and coagulate tissue using thermal energy. Using a laser reduces the risk of floating footplate, consequently reducing the risk of inner ear injury, and allows creating a fenestra that is suitable for the size of the prosthesis. The laser also allows for a bloodless surgical field.

A wide variety of lasers are currently used, both in the visible and invisible light spectrum, with different characteristics. The laser strikes the tissue with a focused beam, producing intense heat and destroying the tissue. The main properties characterizing a laser and therefore determining the laser-tissue interaction are the wavelength, power, and duration of exposure. Depending on the type of laser used, different tissue reactions may occur. The interaction between laser and tissue depends on the degree of energy absorbed by the tissue. If the wavelength is short, absorption occurs by proteins, lipids, and nucleic acid. Infrared wavelengths are mostly absorbed by water, whereas wavelengths in the visible spectrum are mostly absorbed by hemoglobin.

Heat diffusion into the tissue may lead to coagulation, vaporization, carbonization, or melting. Tissue can be cut with precision, causing minimal damage to surrounding tissues. The laser light can be emitted continuously or in pulses. The energy delivered to the tissue surface is measured in joules per square centimeter (J/cm^2^). In stapedotomy, the laser should not penetrate deeply into the perilymph, which would increase its temperature and lead to possible undesirable effects such as hearing loss and tinnitus. Ideally, the laser should be absorbed by bone, causing punctual ablation of the footplate.^185^

Lasers are used not only for fenestration, but also to cut the stapedius tendon and divide the anterior and posterior crus. Other characteristics that may vary according to each laser include spot size (the larger the spot, the greater the energy dissipation), beam visibility, and type of manipulation (coupled to the microscope or handpiece). Beam visibility is an advantage as it does not require a guide. Lasers with an invisible beam require a guiding visible laser beam, which increases the risk of beam misalignment and distortion of the target site. Larger diameter guide beams can also compromise the fine precision work of stapedotomy.^185^

The laser beam may be delivered by a micromanipulator attached to a microscope or by a fiber-optic handpiece. Using a laser attached to the microscope limits the field of view, which makes its use in certain structures such as the anterior stapes crus more difficult. Manipulating this structure without adequate visibility increases the risk of footplate fracture. It may also hamper visualization and lead to footplate perforation in cases of dehiscent facial nerve. Portable lasers allow performing stapedotomy with a microscope or an endoscope.^186^

Argon, diode, KTP, and thulium lasers can be delivered by silica fibers in a handpiece. The CO_2_ laser is absorbed by the silica and is delivered by an articulated arm on the microscope, which increases the chance of misalignment. Handpieces have been recently developed for CO_2_ lasers. The ideal laser should be easy to use and handle, provide good ablation of bone without penetrating too deeply, and be cost-effective.

Argon and KTP lasers have similar wavelengths and are in the visible light spectrum. They are primarily absorbed by pigmented areas such as the vestibular neuroepithelium and are less well absorbed by water, with potential vestibule damage and dizziness. Invisible wavelengths such as the Er:YAG and CO_2_ lasers have different characteristics and other potential adverse effects. The Er:YAG laser is well absorbed by bone, causing explosive ablation and a shock wave in the target tissue. The CO_2_ laser is well absorbed by water and less absorbed in the inner ear but generates heat that could lead to thermal damage. Laboratory and animal tests confirmed this pattern of absorption, but the clinical relevance of these findings is uncertain.^185^, ^186^

Experimental studies with inner ear models compared CO_2_, thulium, and KTP lasers. Thulium and CO_2_ had increased thermal effects beneath the stapes footplate compared with KTP. These 3 lasers generated less noise than the drill. The thulium laser produced large bubbles within the vestibule, and the KTP laser showed less mechanical effect. Thus, the KTP laser has less thermal, mechanical, and sound effects than the other two. Theoretically, the thulium laser would be less safe compared with the KTP and CO_2_ lasers, for example.^186^

Despite variations in wavelength, tissue absorption, and manipulation, there is no evidence to support the clinical advantage of using one laser over the other. Safe parameters of power settings and pulse durations were described in a review by Young et al.^185^

The main advantage of using lasers is the association between high precision and low risk of footplate mobilization as a result of the “no touch” technique. Several noncomparative studies using the traditional technique (microperforator or microdrill) described audiologic results and side effects in laser-assisted surgery.

When analyzing studies that compared conventional surgery with laser surgery, several factors that interfere with the results must be considered. Most studies have a small number of participants. Studies with more participants are needed to assess SNHL. There is also no systematic evaluation of adverse effects: comparisons are made with different types of lasers and prostheses, and the evaluated audiometric frequencies and follow-up time are not consistent. The lack of standardization of treatment, surgical technique, randomization, allocation to treatment groups, and blinding considerably increase the risk of bias.^186^

Although damage to the inner ear by mechanical manipulation is less likely with the use of a laser, possible harmful effects should not be overlooked, such as overheating of the perilymph (CO_2_), acoustic trauma (Er:YAG), and penetration of the brain endothelium (argon and KTP).^187^

Silverstein et al.^188^ compared the results of patients undergoing conventional surgery with consecutive patients undergoing KTP laser surgery. Patients undergoing laser surgery had improved audiologic outcomes compared with patients undergoing conventional surgery (ABG < 10 dB in 91% vs. 72% of patients, respectively). However, laser-treated patients experienced prolonged dizziness and instability, which lasted for 1–3 weeks in 39% of them. Sakamoto et al.^189^ compared patients undergoing conventional technique vs KTP laser-assisted surgery and found similar hearing and vestibular results in both groups.

Arnoldner et al.^190^ compared the clinical results of conventional vs Er:YAG laser-assisted stapedotomy. The occurrence of floating footplate was similar in both groups, whereas accidental stapedectomy was more common in the conventional technique group (8.7% vs. 1.9% in the laser group). Interestingly, the incidence of perilymphatic fistula was more common in patients operated on with the laser. Hearing results were similar between the groups, as well as tinnitus incidence. The authors showed that there may be worsening of bone conduction thresholds in the first days after laser surgery, with recovery in most cases in the first weeks. This worsening may be due to the mechanical trauma caused by the waves generated in the perilymph. Therefore, direct laser application into the opened vestibule should be avoided, and the energy per pulse and total energy administered in the footplate should be limited. Due to worsening bone conduction thresholds, the authors proposed a technique consisting in the combined use of a laser (to thin out the footplate) and a manual perforator (to facilitate perforation).

Hamerschmidt et al.^191^ compared the use of diode laser with the conventional technique in a small group of patients and found no statistically significant difference. In a multicenter retrospective study with a larger sample, De Vito et al.^192^ compared the conventional technique with the use of CO_2_ laser and found similar results in both groups. The group treated with CO_2_ had a higher percentage of patients with an ABG < 10 dB despite lack of statistical significance. Pauli et al.^193^ conducted a retrospective study and found no significant differences in hearing thresholds in patients undergoing different surgical techniques (KTO, CO_2_, and drill). Surgical complications were rare.

In a multicenter retrospective study, Altamami et al.^194^ compared the use of a microdrill with CO_2_ laser and did not find statistically significant differences. In a systematic review, Bartel et al.^195^ concluded that there is no evidence that either laser fenestration or conventional fenestration are superior to each other in relation to hearing outcomes. More than 70% of patients in both groups achieved an ABG < 10 dB. The use of diode laser is more recent and has been investigated in the past years. Current evidence, including a randomized clinical trial, does not show better results with diode laser over the traditional technique.^196^

Over the years, several studies comparing results with and without the use of laser have been conducted. A meta-analysis conducted by Fang^174^ showed better results with the use of laser, although the complication rates were similar. Of 3 studies published after the meta-analysis was conducted, 2 showed better results with the microdrill and 1 showed better results with the laser.

Wegner et al.^197^ conducted a systematic review that showed no difference in immediate postoperative hearing results and vertigo when comparing the use of laser and conventional techniques for fenestration. In this review, some studies with a moderate-to-high risk of bias showed differences in ABG closure and vertigo in the immediate postoperative period that favored the conventional technique, whereas others showed better hearing results with laser-assisted surgery. However, footplate fracture and SNHL were more common in the conventional group when compared with the laser group, whereas tinnitus was more common in the laser group. There was great heterogeneity among studies, which prevented data pooling and required the use of descriptive analysis. Data should be analyzed with caution due to significant risk of bias. SNHL occurred in less than 1% of cases, similar to what was found in a large series of patients undergoing conventional surgery.^132^ Therefore, studies with a much larger population are needed to confirm the superiority of laser surgery, as small samples can overestimate or underestimate the results.

In the absence of evidence to support the superiority of one technique over the other (conventional vs laser), surgeons should choose the technique that they feel more comfortable and safer using. One argument in favor of laser surgery is the reduced technical difficulty in comparison with conventional surgery. Importantly, stapedotomy – both conventional and laser-assisted – should only be performed by experienced surgeons.

Randomized clinical trials with cohorts with a well-established follow-up period and a low risk of bias are needed for more robust evidence-based statements. In experienced hands, both the conventional and laser-assisted techniques present satisfactory results. Consequently, the best approach is the one with which the surgeon feels most comfortable.

Studies comparing results with different types of lasers have significant risk of bias, lack of randomization, inappropriate allocation, and lack of blinding in the analyses. In addition, they differ in terms of laser, technique, audiometric testing, and follow-up time. These factors impair the pooled clinical interpretation of results of several studies.

Kamalski et al.^157^ conducted a systematic review comparing different types of laser. The CO_2_ laser had a slightly better ABG closure compared with the KTP and Er:YAG lasers, and there were no cases of SNHL. However, the clinical relevance of these results is unclear, and risks of bias should be considered before generalizing these findings to clinical practice. Furthermore, differences in hearing outcomes when comparing the use of the KTP and CO_2_ lasers are small and might not be clinically relevant. Differences between the Er:YAG and CO_2_ lasers are more significant and clinically relevant.^198^

Kamalski et al.^199^ also conducted a prospective, uncontrolled clinical trial comparing hearing results with the use of the CO_2_ laser vs the thulium laser. The success rate (defined as an ABG < 10 dB) was lower in the thulium than in the CO_2_ group at 3 and 12 months of follow-up. Patients treated with thulium laser-assisted surgery were also at greater risk of SNHL and tinnitus. Therefore, the use of thulium laser appears to be more damaging to the inner ear compared with the CO_2_ laser.

Szyfter et al.^198^ conducted a retrospective comparative analysis between patients undergoing surgery with CO_2_ and Er:YAG lasers. They did not find significant differences between the groups despite some authors having previously reported a greater risk to the inner ear with the use of Er:YAG.

Randomized trials assessing adverse effects as primary outcomes in laser surgery are lacking. Because SNHL (main adverse effect) is a rare complication in general, studies with larger samples are needed to compare its occurrence in groups of patients operated on with different lasers.

Although the possibility of mechanical injury from trauma is less likely with the use of a laser, it should not be disregarded. The CO_2_ laser could overheat the perilymph and damage the HCs, whereas the Er:YAG laser could cause acoustic trauma, for example. Both complications could cause SNHL.^157^, ^200^, ^201^

Vertigo could be caused by direct damage to the brain endothelium by the KTP or argon laser.^201^ These possible and potentially disabling side effects should be considered when choosing the laser. Previous studies have shown vertigo rates of up to 20% and 19% after Er:YAG and KTP surgery, respectively.^202^

Tinnitus after laser surgery has also not been evaluated as a primary outcome. Case series studies have described tinnitus rates of up to 9% and 4% after CO_2_ and Er:YAG use, respectively.^188^, ^203^

Conclusions on the advantages and disadvantages of different types of lasers are based on a limited number of studies with significant bias. Therefore, the choice of laser depends on the surgeon’s preference, availability, cost, and ease of handling. High-quality randomized clinical trials are needed to reach conclusions with a high level of evidence.

Possible reasons for conductive hearing loss after primary surgery include displaced or fixed prosthesis; subluxated, fixed, or eroded malleus or incus; and fibrosis or regrowth of otosclerotic *foci* in the oval window. Several studies report unsatisfactory results in revision surgeries.^204^

In revision surgery, it is necessary to clean the oval window niche from granulation tissue or other tissues used in primary surgery to visualize the margins of the footplate and to check if the prosthesis is well located. Excessive manipulation may cause SNHL. Inadvertent application of the laser to the prosthesis may cause impacts on the prosthesis if still located in the vestibule opening.^205^

Unlike primary surgery, revision surgery involves removing the soft tissue that fills the middle ear. Therefore, lasers with properties that allow vaporization of bone and soft tissue may be advantageous. Albers et al.^204^ evaluated the outcomes of patients undergoing revision surgery with CO_2_ laser. The footplate was successfully perforated with a single shot in only 22% of patients, whereas in primary surgery this was possible in 70% of cases. ABGs of up to 10 dB and up to 20 dB were observed in 55% and 41% of patients, respectively. Such results are worse than in primary surgery, but superior to revision surgery using the conventional technique.^204^

In a retrospective study of revision surgery with KTP laser, Silverstein et al.^206^ found no statistically significant difference in hearing results between laser surgery and conventional technique. However, an absence of adhesions was noted in laser-assisted primary surgery.

Wiet et al.^207^ compared the efficacy and safety of revision stapedotomy with the use of argon laser or conventional technique. Laser surgery demonstrated statistically significant advantage in both parameters. Therefore, using laser in revision surgery appears to be safe.

Although microscopic stapedotomy is very successful, it has some limitations. Technical difficulties may occur due to EAC abnormalities and anatomical variations of the scutum. In patients with a very narrow EAC, the middle ear may need to be accessed through other routes, such as the retroauricular and transcanal regions, and bone may need to be removed from the EAC.^208^

In microscopic surgery, the surgeon is unable to visualize the anterior crus of the stapes and has to blindly fracture it. The use of the endoscope offers a wider field of view, with greater magnification. However, the surgeon has no 3D view, and its use is associated with a longer learning curve. Unlike in microscopic surgery, bone wall structures do not need to be removed for adequate visualization.^209^

The main lasers used in stapedotomy (CO_2_ and KTP) are contact lasers, potentially useful in one-handed endoscopic surgeries. Contact lasers are more precise and stable for footplate drilling. Kuo et al.^210^ conducted a retrospective study comparing patients who underwent endoscopic stapedotomy with KTP laser vs patients who underwent microscopic stapedotomy. Endoscopic surgery had a longer operating time and, despite requiring less bone manipulation, the surgeons spent more time wiping the endoscope or on hemostasis, as well as setting up the laser. More practice might be needed to reduce operating time.

There were no significant differences in hearing outcomes in both groups, as well as complications. However, this was a comparative, retrospective study with a small sample size, meaning that several confounding factors could have influenced the analysis. Studies with small samples may lead to biased effect estimates.

##### Argon laser

The argon laser was the first laser to be used in stapedotomy. It has a relatively short wavelength (488–514 nm) and is absorbed primarily by hemoglobin (which allows good hemostasis) and less by bone tissue, which has a lot of water in its composition. Low bone absorption can increase penetration and temperature in the inner ear, and clinical experiments found a temperature elevation of up to 10 °C in the perilymph.^186^, ^211^

The argon laser has a visible beam, therefore not requiring a guiding visible laser beam, which reduces the risk of misalignment.^212^, ^213^ Initially, the laser was attached to the microscope, but a fiber-optic microhandpiece was later introduced, leading to an increase in beam diameter (from 100 to 500 micrometers) and a reduction in radiation. The fiber-optic microhandpiece reduced the temperature increase in the perilymph to 2–3 °C and allowed access to structures that are difficult to visualize with the microscope, such as the anterior crus of stapes.^214^ The argon laser proved to be safe if used at low power.^215^

##### CO_2_ laser

The CO_2_ laser has a long wavelength (from 9600 to 10,600 nm) and is primarily absorbed by water. Therefore, this type of beam is well absorbed by bone, which is composed of >50% water. Increased bone absorption protects from deep penetration into the inner ear, reducing the chance of temperature elevation. Despite this, temperature increase and SNHL have been described with the use of argon laser in previous studies.^216^ However, using short pulses limits heating to 0.3‒0.5 degrees, promoting heat dissipation and reducing the chance of inner ear injury.^217^ Several studies have shown good results and low complication rates with the use of CO_2_ laser. It has an invisible beam, which is very precise when used with delicate manipulators, and therefore requires a guiding visible laser beam (usually helium/neon).

“One-shot” or multiple-shot technique can be used. Using a single shot decreases the chance of inner ear injury and requires precise drilling of the footplate (this type of laser is equipped with a system that uses rotating mirrors to precisely focus the laser beam).^218^ The use of multiple shots increases the risk of the vestibule being hit by laser beams when the footplate has already been perforated.^216^ A study by Just et al. showed a trend towards worse bone conduction thresholds at 6 kHz and 8 kHz when more than one laser shot was applied.^219^ The CO_2_ laser was initially used attached to the microscope, but has recently been used in a portable piece, with precise propagation.

##### KTP laser

The KTP laser is only partially absorbed by bone (wavelength of 532 nm), with potential risk of temperature elevation and inner ear injury. However, despite concerns about thermal injury, clinical studies have shown that the KTP laser is safe when used at low power. Vicent et al. reported only 1 case of SNHL in a series of 410 patients (0.25%).^220^ The KTP laser is well absorbed by hemoglobin (which enables good hemostasis), has a visible beam, and can be delivered by a fiber-optic handpiece.^211^

##### Er:YAG laser

The Er:YAG laser has a long wavelength (2940 nm) and is strongly absorbed by bone tissue, thus allowing precise ablation. It minimally penetrates surrounding tissue such as the inner ear and, therefore, does not significantly increase the temperature.^221^

Szyfter et al.^200^ demonstrated good hearing results in patients undergoing Er:YAG laser-assisted surgery followed up for 3 years. There were no cases of SNHL. The authors believe that the complication rate is directly linked to the dose applied during surgery. Thus, the use of the Er:YAG laser is not indicated in cases of advanced otosclerosis, as it would require higher doses.

The laser’s beam is invisible (infrared), and the laser may be used in a fiber-optic handpiece, which allows safe radiation transmission. It is not well absorbed by hemoglobin, with low capacity for hemostasis. The laser pulse generates a sound wave that patients may hear as a gunshot, which is considered a trauma to the inner ear. This may be a disadvantage in patients undergoing stapedotomy under local anesthesia.^222^

##### Diode laser

The diode laser is an electronic laser with a wavelength of 805–980 nm. These wavelengths fall between the absorption peaks of hemoglobin and water and, therefore, are less well absorbed by bone. These lasers are available in portable handpieces and consist of two semiconductors that deliver the laser via quartz fibers and produce infrared radiation. The contact of the laser fibers with the target structure allows greater precision. They have little thermal and mechanical effects, which is an advantage over other lasers. The fiber diameter can be adjusted according to the procedure. They have good clotting ability. After footplate penetration, the excess energy is absorbed by the perilymph in the pigmented region of the brain endothelium. Gerard et al.^223^ retrospectively reviewed the outcome of patients undergoing diode laser stapedotomy. One patient had SNHL and 86% of patients had a postoperative ABG < 20 dB.

##### Thulium laser

The thulium laser is widely used in urology procedures (prostatectomy and lithotripsy) as well as in laryngeal surgery. It has a wavelength of 2013 nm and is primarily absorbed by water, therefore also has good bone absorption (but not as good as the CO_2_ laser). It provides good hemostasis and precision during tissue resection. The laser is delivered by a silica fiber-optic handpiece and requires the use of protective eyewear. As with the Er:YAG laser, it produces a sound wave. Cadaver studies of inner ear models also showed that the laser increases temperature in the perilymph and produces vapor bubbles after its use.^185^ In a retrospective study with a relatively large number of cases, Covelli et al. found improved functional hearing with no signs of inner ear injury, suggesting that the thulium laser is a safe tool for stapedotomy.^224^

#### Recommendations (Box 11)

**Box 11:** Recommendations – Use of lasers and microdrill for stapes surgery.

The microdrill should be used for footplate perforation (Weak recommendation – Moderate-quality evidence).
The microdrill can be safely used for footplate fenestration in endoscopy surgery (Insufficient evidence).
Lasers should be used in otosclerosis surgery (Weak recommendation – Moderate-quality evidence).
Different types of lasers may be used with similar results (Weak recommendation – Low-quality evidence).
Lasers should be used in revision surgery for otosclerosis (Weak recommendation – Moderate-quality evidence).
Lasers should be used in endoscopic otosclerosis surgery (Insufficient evidence).

#### Prostheses: materials, indications, and results

The basic principle of prostheses used in otosclerosis surgery is to achieve a secure connection between the long process of the incus, which has preserved mobility, and the perilymph in the oval window.^225^ More than 100 types of stapes prostheses have been developed since Shea and Treace first carved a stapes replica in Teflon.^225^

Evolutions in surgical technique over the years required prostheses to evolve as well. Initially, the prosthesis had a wider base for sealing the oval window after removal of the stapes footplate. This base was later narrowed into a piston-like shape, which was designed to seal only the fenestra over the stapes footplate, and could be used in combination with different grafts or not.^225^ Changes in surgical materials and the emergence of materials with greater biocompatibility were also important factors in the development of new stapes prostheses over the years, as these new materials allowed to reduce the force required to adequately place the prosthesis on the target site. Shape-memory prostheses have also been developed.^225^, ^226^

Although the success rate of stapedectomy in 1960 was in the 90% range, some challenges persisted, such as necrosis of the long process of the incus (particularly with the polyethylene strut), postoperative dizziness, loosening of the wire loop, fistula, and granuloma formation with Gelfoam prostheses.^225^ Other issues related to patient rehabilitation such as previous erosion of the long process of the incus or absence of incus, where the stapes prosthesis should connect the fenestra to the malleus, prompted the development of alternatives to treat each case individually.^225^ There are several models currently available on the market, which vary in shape, weight, diameter size, site, anchorage, and material.

##### Types of prosthesis

Several types of prostheses have been developed since the one originally designed by Shea in 1955. Prostheses can vary in size, diameter, shape, and material. Fritsch and Naumann proposed a classification of stapedotomy prostheses into four categories: wire loop, piston, bucket, and homemade.^227^ Of the 3 major commercial types (wire loop, piston, and bucket), each prosthesis can be divided into 3 anatomic regions: the incus attachment end, the shaft, and the oval window attachment base.^227^

Regarding the incus attachment end, evolutions in surgical technique and postoperative complications led to innovations in how to keep the prosthesis fixed on the long process of the incus without resulting in incus necrosis.^227^ Necrosis of the long process of the incus is secondary to ischemia due to pressure applied with a special forceps to close this end of the prosthesis, insufficient crimping, or foreign body reactions.^227^

Regarding crimping, stapedotomy prostheses can be divided into self-crimping and those requiring manual crimping. Such complications culminated in the development of alternatives such as Teflon and shape-memory prostheses as well as nitinol prostheses, which return to their predefined shape by memory effect or after exposure to a heat source, without the need to crimp the prosthesis in the long branch of the incus, considered one of the most delicate moments in ear surgery in general.^226^, ^228^ Of note, the need to expose nitinol prostheses with thermal memory to a heat source close to the incus, as well as the possibility that the initial memory position is too tight for the diameter of the long process of the incus in certain patients, was associated with a possible worse audiologic outcome and the possibility of complications such as those previously described.^125^

The shaft underwent multiple changes over the years. Wire loops commonly use a 36-gauge shaft,^227^ whereas in pistons the shaft has the same diameter from the base to the incus attachment end.^227^

As for the oval window attachment base, several models have also been proposed over the years, with different shapes according to the proposed surgical technique (stapedectomy or stapedotomy), with or without the placement of a graft over the oval window.^227^ Because stapedotomy is performed more frequently, piston diameters changed from 0.3 to 0.8 mm, and some bases have measuring notches to measure depth of incursion.^227^ Due to physical phenomena, prostheses with a larger diameter and composed of impermeable material are known to have better sound conduction.^227^ Regarding the total size of the base, some prosthesis have a predefined size and need to be measured intraoperatively to choose the appropriate model, whereas in others the base can be trimmed to the desired length and are manufactured as a “one-size-fits-all” design.

Some situations may require the use of uncommon prostheses for auditory rehabilitation. Patients with erosion of the long process of the incus or other ossicular chain disorders that preclude adaptation to conventional stapes prostheses may benefit from the use of a prosthesis that can adapt to the remainder of the long process or from a malleostapedotomy.^125^, ^229^, ^230^ Prostheses used in the remainder of the long process are specially adapted for fitting, and may include crimping or spiral-shaped models.^125^ The malleus prosthesis has a longer shaft that connects the manubrium to the fenestra on the stapes footplate.^125^, ^229^ Importantly, the choice of malleus prosthesis for malleostapedotomy should be individualized, and surgeons should consider the distance between the malleus and the oval window and the angle formed by these two structures.^230^

##### Materials

Stainless steel is one of the most popular materials in the manufacture of stapes prostheses due to its rigidity, ability to maintain its shape, and fixation to the incus or malleus. It also has adequate malleability for performing surgery and can be molded and cut.^125^ There are 2 variants of stainless steel commonly used in medicine, the 300 and 400 series, which have different characteristics.^225^ The 300 series is typically used for implantable systems and is composed of chromium, carbon, nickel, and manganese. The microdipoles are arranged randomly, reducing its magnetism, and these systems can be safely exposed to magnetic fields of up to 9.4 Tesla.^125^, ^225^

Platinum was suggested as a good option due to its malleability, but its use was associated with a higher occurrence of necrosis of the long process of the incus.^125^ This increased rate of necrosis is believed to be associated with local toxicity or alterations in incus attachment.^125^ Platinum prosthesis can be safely exposed to magnetic fields of up to 1.5 Tesla.^231^

Titanium is considered a good material for vibration conduction because it is lightweight and rigid.^125^ Another advantage is that after oxidation, a protective layer of titanium oxide is formed on the titanium metal surface, increasing its biocompatibility.^125^ Titanium is nontoxic to the human body and cannot usually trigger an immune response, presenting reduced granulation and scar tissue formation compared with Teflon and gold prostheses.^125^ Titanium prostheses can be safely exposed to magnetic fields of up to 3.0 Tesla.^231^

Another option are nitinol prostheses (alloys of titanium and nickel), which return to their original shape when heated.^225^, ^228^ The main complications of nitinol prostheses include vestibule displacement and insufficient fixation to the long process of the incus, which can be resolved with additional crimping to ensure adequate adhesion. Teschner et al.^228^ explained that in case of insufficient fixation, as long as the prosthesis is not dislocated, local fibrous reactions are sufficient to fixate it and achieve good audiologic outcomes. Nitinol prosthesis are safe for MRI use and do not move when exposed to magnetic fields of up to 1.5 Tesla.^228^, ^231^ Regarding biocompatibility, most studies show that these alloys have low cytotoxicity and low genotoxicity, in addition to having adequate corrosion properties, with negligible release of Nickel ions.^228^

Teflon is among the most common materials, consisting of a polymer with a low coefficient of friction, chemically stable, malleable, and resistant to corrosion.^125^ It has the advantage of having a ‘memory effect’, reducing the chance of complications related to necrosis of the long process of the incus due to ischemia.^232^ Teflon does not have ferromagnetic properties, therefore it is safe for MRI use.^125^, ^231^

Regarding audiologic outcomes and postoperative complication rates, Bansal^233^ found no differences between Teflon and titanium prostheses, which were considered equivalent. Teschner et al.^228^ assessed hearing outcomes in patients undergoing stapedotomy with a Teflon-platinum prosthesis vs superelastic nitinol prostheses and obtained equivalent results with both prostheses. Regarding piston diameter, several studies show a trend towards better hearing results with larger diameter prostheses.^225^, ^234^, ^235^

The surgical outcome of malleostapedotomy depends on the severity of the case, the skill of the surgeon, and the choice of the appropriate type of prosthesis.^230^, ^236^ In general, malleostapedotomy is considered a safe procedure, with 41.2% of patients achieving an ABG < 10 dB and 70.6% achieving an ABG < 20 dB.^236^ However, it requires the use of a longer prosthesis that bypasses the ossicular chain, which is thought to be responsible for protecting the inner ear from pressure variations, thus making the inner ear more susceptible to injury.^229^

#### Recommendations (Box 12)

**Box 12:** Recommendations – Prosthesis for Stapes surgery.

No prosthesis material is superior to another in stapedotomy regarding hearing outcomes (Strong recommendation – High-quality evidence).
Before allowing a patient to undergo an MRI examination, the prosthesis material must be identified, especially in patients who underwent surgery in the past (Strong recommendation – High-quality evidence).
In general, no prosthesis currently available on the mark is superior to another in terms of model and material, and attention should be paid only to possible specific indications according to anatomical alterations in primary and revision surgery (Strong recommendation – High-quality evidence).

#### Postoperative care

Just as there are variations in stapes surgery technique, there are also variations in postoperative management. Once considered an inpatient procedure, stapedotomy in the US has evolved into an outpatient, or a 23-h inpatient procedure. Outside the US, many centers believe that it is important to admit the patient after surgery. Although stapes surgery is considered a clean otologic surgery, a Cochrane report found no evidence to support the perioperative use of antibiotic therapy.^237^ Most centers continue to treat patients with antibiotic prophylaxis because the risks associated with postoperative infection include deafness and labyrinthitis.^238^ In addition, intraoperative and postoperative corticosteroids can be used to minimize the chance of serous labyrinthitis. However, clinical studies to support this are lacking.

#### Complications in stapes surgery

Stapedotomy is usually a safe procedure, with good results, few complications, and a failure rate of approximately 6%.^132^ Surgical complications are uncommon, may occur intraoperatively or postoperatively, and can include the following (Table 7).

**Table 7:** Complications in stapedotomy.

Intraoperative	Postoperative
Bleeding	Profound deafness
Tympanic membrane perforation	Necrosis of the long process of the incus
Chorda tympani nerve injury	Labyrinthitis
Facial nerve injury	Peripheral facial paralysis
Pneumolabyrinth	Dysgeusia
Perilymph oozer or gusher	Vertigo
Floating footplate	Conductive hearing loss
Incus subluxation	

Surgical failure usually results from poor positioning or inadequate length of the prosthesis. Due to the progressive nature of the disease, 20% of patients will need revision surgery.^239^, ^240^

Disease progression or cochlear involvement cannot be predicted. After stapedotomy, hearing loss can progress at variable and unpredictable rates.^241^ A study evaluating patients 30 years after stapedectomy found that 88% had bilateral otosclerosis and 66% had moderate to profound loss secondary to the progressive development of SNHL.^242^

According to Strömbäck et al.,^243^ 90% of patients were satisfied with the hearing improvement 1 year after the surgery. However, the complications associated with stapedotomy, although uncommon due to advances in the technology of PSAPs, require that surgical indication and the chance of failure be thoroughly discussed with the patient during preoperative evaluation.

Some reasons for surgical failure may be observed or suspected during the diagnostic investigation, before the surgical procedure. History of progressive hearing loss since childhood may suggest malformations such as an enlarged vestibular aqueduct, whereas aural fullness and pressure-induced vertigo may be indicative of superior semicircular canal dehiscence.^244^

##### Intraoperative

In addition to stapedotomy-related complications, other situations that may increase the risk of surgical failure or even complications may occur during the procedure.

###### Bleeding

In addition to patient history and preoperative exams that assess coagulation disorders, positioning the patient with the head elevated in relation to the body and injecting an anesthetic solution with a vasoconstrictor a few minutes before starting the procedure is essential to prevent bleeding. If bleeding persists, it may be controlled using a cotton pledge or an absorbable hemostatic gelatin sponge soaked in epinephrine. The bleeding should be controlled before opening the oval window, as placing the prosthesis into a stapedotomy in the presence of bleeding is significantly more difficult due to the risk of aspiration in the opened region. In addition, some studies have shown that the presence of blood in the vestibule has deleterious effects.^245^

###### Tympanic membrane perforation

Tympanic membrane perforation may occur during detachment of the tympanomeatal flap at the end of the surgery. If the defect is small, a piece of absorbable hemostatic gelatin sponge may be placed on the region to assist in wound healing. If the defect is larger, the tympanic membrane should be reconstructed using a temporalis fascia graft when using the Lempert access or a perichondrium or tragus cartilage graft when using the endaural access.

###### Peripheral facial paralysis

The tympanic segment of the facial nerve is the most prone to dehiscence of its bony canal and passes beside the oval window in the middle ear. During stapedotomy, it is more susceptible to trauma by manipulation, aspiration, or even by the use of a topical anesthetic or vasoconstrictor. Identification of the facial nerve at surgery is essential. If dehiscent, special care should be taken, especially when drilling the footplate. Sometimes the nerve is partly covering the stapes footplate, which requires using a microdrill on the lower edge of the window to enlarge the space, allowing placement of the stapedotomy prosthesis. When the footplate is completely covered by the facial nerve or when there is a bifurcation of the nerve involving the stapes suprastructure, surgery is contraindicated. Facial nerve injury can also occur due to PSA injury due to ischemia.^144^

###### Incus luxation

Incus luxation mostly occurs during curettage of the external acoustic meatus or when attempting to secure the prosthesis on the incus. Preventive measures include using a microdrill to remove excess bone from the posterior wall of the external acoustic meatus or using the reverse technique, in which the prosthesis is placed before the incus-stapedial disarticulation, maintaining a more fixed structure at the time of positioning between the footplate and the incus.^153^ If luxation occurs, the prosthesis should be placed using the conventional technique, sometimes with both hands, with an instrument that supports the incus while the prosthesis is being placed.

###### Obliterative footplate

Obliterative footplate is present in 3% of cases of otosclerosis, increasing the risks of surgical failure and complications such as SNHL (4.8%) and perilymphatic fistula (2,4%). It also increases technical difficulty, requiring the use of a microdrill or laser to perforate the footplate. In these cases, high-resolution CT may help to identify footplate thickening, which is important in surgical planning to ensure that the necessary material is available to proceed with the procedure.

###### Floating footplate

The footplate may be detached from the annular ligament at the time of fenestration, becoming very mobile and hard to completely perforate. In these cases, the surgeon should not try to remove the footplate, as it may completely penetrate the vestibule, increasing the risk of SNHL. As with incus luxation, using the reverse technique helps to maintain a more rigid structure, reducing the force exerted on the footplate at the time of fenestration and decreasing the risk of floating footplate.^153^

###### Perilymph gusher

Brisk perilymph (Cerebrospinal Fluid [CSF]) flow under pressure after perforation of the footplate is common in cases of malformations such as enlarged vestibular aqueduct and dysplasia of the internal acoustic meatus and cochlea, which can sometimes be identified by an imaging exam prior to the procedure. If gusher occurs, a vein, fascia, or fat tissue can be placed over the window and the prosthesis may be placed in the usual fashion. In the postoperative period, a collection bag should be used to assess whether there is fluid coming out of the ear. The patient should remain at absolute rest, with the bed headboard elevated, and should receive medication such as acetazolamide to reduce CSF production flow. In these cases, the risk of SNHL is high.

###### Corda tympani nerve injury

The chorda tympani nerve, responsible for taste perception in the anterior two-thirds of the tongue, needs to be displaced to allow complete visualization of the oval window and space for the surgical procedure; however, it should not be cut. In general, taste alteration, especially metallic taste, is the second most common complaint in the postoperative period of stapedotomy, and may be present in up to 60% of cases in the immediate postoperative period and 5% after 1 year of the procedure.^193^ In bilateral surgery, extra care should be taken when operating the second ear, especially if the surgeon does not know whether the chorda tympani nerve was cut on the first operated ear, increasing the risk of dysgeusia.

###### Pneumolabyrinth

A small amount of air is commonly found in the labyrinth after fenestration. Aspiration or application of a hemostatic sponge on the window should be avoided, as they may lead to loss of perilymph and increase the risk of postoperative SNHL.

##### Postoperative

###### Infection

Postoperative infections are rare. When they occur, they typically affect the outer ear and may be treated with antibiotic ear drops. The use of antibiotic prophylaxis was not shown to be necessary in stapedotomy.^246^

###### Vertigo

Otosclerosis-associated vertigo is a common symptom. Dizziness or imbalance is very common and expected in the immediate postoperative period, lasting from hours to a few days. However, disabling and long-lasting vertigo may be related to greater intraoperative manipulation, dry vestibule, or a long prosthesis or a prosthesis in an anterior position stimulating the saccule. In these cases, antivertigo drugs should be used; if the patient does not improve, the CT may identify a long or dislocated prosthesis within the vestibule. If the patient still does not improve, surgical revision for replacing the prosthesis with a shorter one may solve the problem. Other causes of postoperative dizziness may include BPPV and perilymphatic fistula.

###### Labyrinthitis

After surgical manipulation of the ear, the healing process involves a low level of serous labyrinthitis, which may be responsible for complaints of dizziness in the first postoperative days. In some patients, dizziness significantly worsens after 1 week and may be accompanied by worsening hearing acuity. In these cases, treatment with corticosteroids should be started and, as it is not possible to rule out bacterial infection, the use of antibiotics is also recommended.^244^ Imaging is necessary to assess cochlear permeability and the appearance of ossification.

###### Sensorineural hearing loss

Severe SNHL affects 0.5%–2% of patients undergoing stapedotomy. High-frequency SNHL is common and may be transient and is mostly associated with manipulation, drilling, and aspiration. Low-frequency SNHL in association with EH has been reported in up to 10% of patients after stapedectomy.^244^

###### Conductive hearing loss

Conductive hearing loss should be thoroughly assessed. If hearing acuity does not improve postoperatively, possible reasons include a short prosthesis, malleus or incus fixation, oval window obliteration due to otosclerosis, and superior semicircular canal dehiscence. However, if hearing acuity improved initially and then worsened again, possible reasons include prosthesis displacement and necrosis of the long process of the incus, which account for 34% of revision surgeries.^132^ Imaging and, if necessary, revision surgery should assist in the differential diagnosis.

###### Peripheral facial paralysis

In addition to the risks of facial nerve injury previously described, peripheral facial paralysis may occur days after the surgery and is usually associated with reactivation of varicella zoster or herpes simplex viruses during manipulation. Treatment with corticosteroids and antivirals is indicated in these cases.

#### Revision surgery

Revision surgery is indicated in up to 20% of cases of primary otosclerosis surgery when there is persistent or recurrent ABG ≥ 20 dB, intractable vertigo, or SNHL with suspected perilymphatic fistula or granulation tissue.^247^, ^248^, ^249^, ^250^ Symptom onset may occur early, such as persistent hypoacusis, vertigo, or SNHL typically associated with intense tinnitus, or they may appear later in a sudden, fluctuating, or progressive manner, such as recurrent ABG. Except in cases of suspected perilymphatic fistula or granulation tissue, which according to some authors should be treated early,^251^ an observational period of 6 weeks^252^ to 3 months^253^ is recommended. Because the outcomes of revision surgery in the literature are inferior to those of primary surgery, its indication should be carefully evaluated.^112^, ^252^

The success rate of ABG closure ≤10 dB is 35%–80%, whereas recent studies have reported lower rates of SNHL (>15 dB), ranging from 0% to 2.7%. Blijleven et al.^248^ and Schwam et al.^240^ found SNHL rates of 5% and 13.1%, respectively, but used a threshold increase of 10 dB instead of 15 dB as a criterion. In Sweden, Lundman et al.^254^ obtained inferior results compared with results from large centers, which the authors believe may be associated with the smaller number of procedures performed at their center. They suggested that patients should be referred to more experienced centers and that results from large centers should not be extrapolated to the local reality of small centers when advising patients. Jervis-Bardy et al.^255^ investigated 15 patients aged <20 years to evaluate revision surgery in the pediatric population. The results were similar to those obtained in the general population, with no cases of significant SNHL. Lippy et al.^239^ evaluated 120 patients aged ≥65 years and obtained results similar to those of a control group consisting of patients aged <65 years, also indicating that age is not an isolated factor for higher risks or contraindication to revision surgery.

Although commonly performed with the microscope, Fernandez et al.^249^ conducted an uncontrolled retrospective study of endoscopic revision surgery and found similar results. Iannella et al.^256^ evaluated a series of 6 patients undergoing malleostapedotomy as revision surgery with the use of an endoscope and found comparable results to studies using a microscope. Bernardeschi et al.^247^ found that rhinologic disease was significantly more frequent in patients undergoing revision stapes surgery compared with primary surgery, and this difference was not addressed by other authors. Recent publications mention the indication of revision surgery for persistent or recurrent persistent ABG ≥ 20 dB and intractable vertigo,^112^ but not for SNHL.^7^, ^8^, ^112^, ^252^ However, only a few studies investigated intractable vertigo as an indication for revision surgery; those that did found complaints of vertigo in 2%–3% of cases, mostly due to the prosthesis being too long. Patients responded well to replacement with an appropriately sized prosthesis.^239^, ^248^, ^252^

Persistent ABG ≥ 20 dB may indicate incorrect technique in the primary surgery, lateral fixation of the malleus or incus to the attic, or the presence of a previously undetected third window, usually leading to worse results in revision surgery.^112^, ^252^, ^253^ Recurrent or increasing ABG may indicate erosion of the long process of the incus, prosthesis displacement, inadequately sized prosthesis, scar adhesions, ossification of the fenestra, or granuloma.^247^, ^251^, ^253^ In up to 82% of cases, there is necrosis of the long process of the incus and/or prosthesis displacement.^253^ Massimilla et al.^257^ investigated 21 patients with incus erosion who either received a new prosthesis placed proximally to the long process or underwent incus reconstruction with bone cement. ABG was reduced to ≤10 dB in 59% of cases and to ≤20 dB in 86.4% cases, with no cases of SNHL. In cases of erosion of the long process of the incus, incus reconstruction with bone cement, positioning a new prosthesis proximally to the long process when possible, or attaching the prosthesis directly to the malleus are good options for achieving satisfactory results with the different surgical techniques. Adhesions and granulation tissue should be removed, and an appropriately sized prosthesis should be used.^252^, ^253^ Fat, vein, and blood grafts are used around the prosthesis to prevent the occurrence of fistula.^257^

In cases of significant erosion of the long process of the incus or incus/malleus fixation to the attic, the prosthesis may be attached from the malleus to the oval window (malleovestibular prosthesis). Gargula et al.^258^ used a nitinol prosthesis in 12 patients, of whom 10 were undergoing revision surgery. An ABG ≤ 10 dB was achieved in 75% of cases and an ABG ≤ 20 dB was achieved in 92%, with no cases of SNHL. Hudson et al. (Hudson, 2014, Revision stapedectomy with bone cement: are results comparable to those of standard techniques?) used hydroxyapatite bone cement to reconstruct the incus of 27 patients. ABGs of ≤10 dB and ≤20 dB were achieved in 77.8% and 96.3% of cases, respectively, with no cases of SNHL. The results were similar to those achieved with the malleovestibular prosthesis. The use of a laser to open the footplate and lyse adhesions and/or the use of a microdrill to open the footplate are recommended for reducing the risk of SNHL (Sakano, 2022, Revision Stapes Surgery; Hudson, 2014, Revision stapedectomy with bone cement: are results comparable to those of standard techniques?).

Granuloma may occur 7–15 days postoperatively after primary surgery, with SNHL and worsening imbalance occurring in 0.1% of stapedectomies and 0.07% of stapedotomies. There is no consensus on whether to perform revision surgery to remove the granuloma and replace the prosthesis, with concurrent antibiotic use, or whether to simply treat the patient with systemic corticosteroids instead of performing surgery.^8^, ^9^ Granuloma has not been addressed by the most recent studies, except for the ones conducted by Schwam et al. (Schwam, 2021, Outcomes in Revision Stapes Surgery), who reviewed 170 revision surgeries and found granulomas in 2.4% of cases, and Ghazi et al. (Ghazi, 2021, Post-stapedotomy reparative granuloma following use of acellular porcine small intestinal submucosa),who reported granuloma formation with the use of a porcine acellular matrix. Care should be taken when choosing or using tissues around the prosthesis due to the risk of developing granuloma. (Sakano, 2022, Revision Stapes Surgery; Schwam, 2021, Outcomes in Revision Stapes Surgery) (Ramaswamy, 2018, Revision Surgery for Otosclerosis).

In revision surgery, the opening of the oval window, the position of the prosthesis in the oval window and on the incus, the size of the prosthesis, the mobility of the malleus and incus, and the presence of granulation tissue and adhesions should be routinely checked. (Ramaswamy, 2018, Revision Surgery for Otosclerosis) (Polony, 2022, Revision Stapedotomies: The Role of Periprosthetic Scar Tissue Formation in the Development of Unsatisfactory Hearing Results after Stapedotomy) (Wegner, 2018, An internally validated prognostic model for success in revision stapes surgery for otosclerosis).

Preoperative CT can help diagnose the cause of the alteration, although it should be noted that it can overestimate the penetration of the prosthesis into the vestibule. Bernardeschi et al.^247^ showed that CT has good sensitivity for detecting malleus fixation and prosthesis displacement, but low sensitivity for detecting changes in the incus.

Wegner et al. (Wegner, 2018, An internally validated prognostic model for success in revision stapes surgery for otosclerosis) analyzed 705 cases of otosclerosis through multivariate analysis and established an internal mathematical model to predict the chance of success in revision surgery. With 57.7% of cases with an ABG < 10 dB, they identified that the technique used in primary surgery (stapedotomy), the cause of failure (displaced prosthesis or ankylosis of the incudomalleolar joint), and the type of prosthesis used in revision surgery (incus-oval window) were predictive factors of success. Conversely, Bernardeschi et al.^247^ found no differences between patients undergoing stapedotomy or stapedectomy as primary surgery.

Key data from some of these studies are summarized in Table 8.

**Table 8:** Revision surgery results regarding ABG, SNHL, and intraoperative findings.

Reference	N	ABG ≤ 10 dB	ABG ≤ 20 dB	SNHL > 15 dB	Incus erosion	Displaced prosthesis
Bernardeschi et al. (2018)^247^	102	60%	85%	2%	42.16%	20.6%
Blijleven et al. (2019)^248^	66	38	80	5%^b^	5%	27%
Fernandez et al. (2019)^3^	34	68.5%	89.5%	0%	32%	26%
Kanona et al. (2017)^4^	49	80	91	2%	12%	8%
Luryi et al. (2022)^5^	150	65.9	93.8	2.7%	38%	43%
Schwam et al. (2021)^10^	170	40.2	78.1	13.1%^b^	43.4%	24.5%
Lundman et al. (2020)^11^	254	35	69	2.3%	35.6%	48.2%
a Massimilla et al. (2021)^6^	21	59	86.4	0%	100%	‒
a Ianella et al. (2018)^14^	6	33.3	50	0%	0%	0%
a Gargula et al. (2020)^16^	12	75	92	0%	75%	0%
a Hudson et al. (2014)^17^	27	77.8	96.3	0%	100%	‒

N, Number of operations; ABG, Air-Bone Gap; SNHL, Sensorineural Hearing Loss.

a Studies using exclusively malleovestibular prostheses.

b SNHL > 10 dB as a criterium.

#### Recommendations (Box 13)

**Box 13:** Recommendations for stapes revision surgery.

Revision surgery is indicated for recurrent hearing loss with an increased ABG (Strong recommendation – Moderate-quality evidence).
Revision surgery is indicated for persistent hearing loss (Weak recommendation – Moderate-quality evidence).
Revision surgery is indicated for intractable vertigo (Strong recommendation – Low-quality evidence).
Revision surgery is not indicated for SNHL (Strong recommendation – Moderate-quality evidence).

### Nonsurgical treatment

#### Personal sound amplification products

Hearing aids are a good alternative for patients who are not candidates, are unwilling, or have bone conduction thresholds that limit the hearing gain from stapes surgery. PSAPs allow good functional gain for most patients, mainly for those with normal bone conduction thresholds. However, they have limited indications for patients with outer ear disorders such as eczematous external otitis. Although technical evolution has mitigated the effects of occlusion and feedback, they can still make it difficult for patients to adapt to hearing aids. Hearing aids can be customized to amplify only the frequencies needed based on the patient's audiometry. As otosclerosis progresses, additional amplification adjustments may be required.

Although PSAPs are beneficial for patients with otosclerosis, maintaining (particularly batteries) and replacing devices that become obsolete over time leads to accumulating costs over the years. The cost of hearing aids varies greatly; they can be very expensive and are often not covered by health insurance. In addition, the disease can affect children, which significantly increases costs over time considering life expectancy, and patients should not engage in water activities while using the devices.

Cost-effectiveness models may be used to determine the lifetime costs and benefits of certain interventions and compare them against each other. They incorporate both initial costs and years, as well as health-related quality of life to determine the overall value of an intervention. Gillard et al.^259^ argue that, from the patient’s perspective, stapedectomy is a good, cost-effective strategy for the treatment of otosclerosis because it maximizes quality of life and minimizes costs. Probabilistic sensitivity analysis showed that stapedectomy was cost-effective compared with hearing AIDS 99.98% of the time, even when considering revision surgeries. Thus, stapedectomy is a great public health strategy.

#### Drug treatment

Advances in the knowledge of metabolism in inflammatory bone diseases have overcome the well-established barriers of endocrine regulation between bone resorption/reposition and reached an understanding of a local system of regulation of osteoclasts/osteoblast activity mediated by well-described inflammatory cytokines for arthritis. There is evidence of an imbalance in this process in inflammatory bone diseases such as osteoporosis and arthritis, and by extension otosclerosis, which is studied according to new concepts in osteoimmunology.^260^

Modern concepts of the bone remodeling process established the crucial role of a balance between bone formation and resorption in this process, which result from a metabolic balance that is ultimately derived from the effector activity of osteoclasts and osteoblasts.^260^ The inflammatory process in otosclerosis promotes an imbalance in the affected ear and is linked to the production of cytokines that directly influence cell activity.^261^

Medications that target substances produced in the otosclerotic focus, which feed the inflammatory and bone remodeling processes, seem promising for future off-label use via intratympanic delivery in clinical research based on randomized and placebo-controlled clinical trials with a sufficient sample size to demonstrate or not the potential effects of this class of drugs, which directly interfere with the pathogenesis.

##### Sodium fluoride

Sodium fluoride has been empirically used since 1964, initially based on prior knowledge of the similarity between otosclerosis and some collagen 1A1 synthesis disorders, in which there is increased formation of sulfated glycosaminoglycan due to increased activity of the DTDST enzyme.^262^ It was not until 2003 that Grayely et al.^43^ demonstrated this process and its inhibition using sodium fluoride in cultured stapes cells. The mechanism of enzyme inhibition was demonstrated through reduced sulfate uptake in cell cultures, indicating inhibition of enzyme activity in osteoclasts. There are only a few well-designed studies published, and randomized clinical trials are lacking. Bretlau et al.^262^ assessed the effect of sodium fluoride in patients with otosclerosis in a randomized, placebo-controlled clinical trial and found that it was beneficial when administered in doses of 40 mg daily. However, chronic sodium fluoride use (>6 months) has serious renal, hepatic, and cardiovascular side effects. Reports of dysostosis and spinal obliteration are not uncommon.^261^ Currently, sodium fluoride only has historical value, and its use has been limited not by side effects, but by its low therapeutic potential. The low level of evidence in the literature is due to the paucity of impact studies, therefore its use is not recommended.

##### Bisphosphonates

Bisphosphonates can be used in metabolic bone diseases, such as Paget’s disease, and are a first-line therapy for osteoporosis.^263^ They also have a considerable number of adverse effects, such as gastroesophageal irritation, fever, myalgia, and hypocalcemia, and other potential long-term effects such as osteonecrosis of the jaw, atrial fibrillation, and fractures.^264^ The use of bisphosphonates in otosclerosis has not been widely established, but case reports have demonstrated stabilization and even improvement of hearing results in patients with otosclerosis. By interrupting endochondral bone resorption, bisphosphonates offer a solution to the complex remodeling process seen in otosclerosis.^263^, ^264^

A retrospective review did not show significant improvement or deterioration in audiologic outcomes after 6 months in patients treated with alendronate sodium, sodium fluoride, or placebo. Although these results could suggest stabilization of the disease, this effect is unclear because similar results were seen in participants taking placebo.^265^ At the same time, data from gadolinium-enhanced MRI scans demonstrated objective radiological improvement in the oval window region in patients taking alendronate sodium compared with placebo and sodium fluoride. These macroscopic improvements in the most commonly affected site demonstrate that bisphosphonate therapy can alter the remodeling process seen in otosclerosis. Long-term data are needed to verify whether these findings manifest as clinically relevant outcomes, such as hearing stabilization, compared with matched participants taking placebo.

At 12 months of follow-up, Kennedy et al.^266^ detected a small improvement in the audiometric results of patients treated with etidronate compared with placebo, but it was not statistically significant.^266^ However, the follow-up time was a major limitation of the study. Quesnel et al.^265^ conducted a retrospective review and found hearing stabilization at 13 months in patients with progressive SNHL treated with zoledronate or risedronate. Jan et al.^267^ demonstrated the same results in 13 out of 14 ears analyzed and followed-up for 5–9 years, suggesting that bisphosphonates may play a role in the stabilization of hearing thresholds in patients with otosclerosis and worsening SNHL.

There is a lack of consensus on the optimal bisphosphonate, route of administration, duration of treatment, and indication for use in the treatment of otosclerosis. First-generation bisphosphonates such as etidronate, used by Kennedy et al.^266^ in their study, have been largely replaced by third-generation bisphosphonates such as alendronate sodium due to superior antiresorptive properties and reduced effect on bone demineralization.^268^ Among third-generation bisphosphonates, alendronate sodium and risedronate are administered orally and zoledronate is administered intravenously. Stabilization of previously worsening SNHL was observed 5 years after treatment with zoledronate.^265^, ^267^ Similar long-term antiresorptive effects of zoledronate have been demonstrated in patients with osteoporosis, providing a potential single-dose treatment alternative to long-term oral administration.^269^ Data for intracochlear administration of bisphosphonates from animal and cadaver studies are ongoing and may offer a new administration route in the future.^270^

Treatment duration is influenced by clinical response and potential side effects and is guided by a multidisciplinary approach with otolaryngologists and rheumatologists. The side effect profile of bisphosphonates has a considerable influence on duration due to the frequency and potential severity reported among patients with osteoporosis after short- and long-term use.^270^ The risk of side effects should be balanced with the potential risks of SNHL. Bisphosphonates have been well tolerated during the treatment period, with only mild side effects including nausea, vomiting, and headache. The absolute and relative indications for the use of bisphosphonates for otosclerosis are currently unclear and require further long-term evaluation of more robust randomized clinical trials. In the setting of worsening SNHL, data from retrospective studies have demonstrated hearing stabilization, which can be a starting point for further evaluation.

Outcome measures are important for monitoring disease progression in the setting of clinical trials. There is no current outcome measure to objectively assess active disease progression or therapeutic response in patients with otosclerosis. In clinical practice, this is done by evaluating the patients’ subjective symptoms and audiometric results. Optimal audiometric data involve bone conduction and speech recognition thresholds to establish cognitive hearing loss and SNHL progression.^266^ Radiological monitoring of response to treatment is one of the methods for monitoring outcomes, but it needs to be correlated with clinically relevant data. If this method is used, periodic patient control including quarterly renal function and bone metabolism examination with serum calcium and alkaline phosphatase dosage is recommended.

##### Vitamin D

Vitamin D is a coenzyme involved in the regulation of calcium concentration and bone metabolism. It is synthesized from steroid derivatives into an inactive compound in the liver and skin and is transformed into a hydroxylated compound through the action of ultraviolet rays from the sun. In the kidneys, it undergoes further hydroxylation, transforming itself into an active compound.^271^

The association between vitamin D deficiency and autoimmune and inflammatory diseases has been reported for years in the scientific literature. Brookes et al.^272^, ^273^ have reported on the association between otosclerosis and hypovitaminosis D, whereas SNHL has been addressed by other authors. However, vitamin supplementation still lacks support from controlled studies with a high degree of scientific evidence.

Approximately 21.6% of patients with otosclerosis have vitamin D deficiency.^273^ Vitamin supplementation associated with calcium administration can benefit these patients by promoting significant anti-inflammatory activation and stopping disease progression. There have also been reports of substantial improvement in hearing thresholds in 3 out 16 treated patients.^261^ Replacement is advised in cases of hypovitaminosis, with a high degree of recommendation, mainly to reduce the progression of otosclerotic disease.

##### Steroidal anti-inflammatory drugs – Intratympanic corticosteroids

Because otosclerosis may have an autoimmune origin, triggered by chronic measles virus infection, the use of glucocorticoids could be indicated in the treatment of the disease. Glucocorticoids bind to high-affinity cytoplasmic receptors and decrease the production of pro-inflammatory cytokines, thus reducing the inflammatory process. Glucocorticoids reduce the activity of the sulfate transporter protein, which sulfates glycosaminoglycans, facilitating bone turnover in otosclerosis.^43^

Few authors have investigated their use in otosclerosis, and the scarce publications on this topic mostly refer to the perioperative use of methylprednisolone in otosclerosis, in addition to the small sample sizes and short-term evaluations.^274^ Chronic steroid use is associated with important side effects, such as diabetes and osteoporosis, among others. Long-term use of this drug class for the treatment of otosclerosis lacks further research and, therefore, is not recommended for treatment.

A promising alternative is the intratympanic use of corticosteroids, which would restrain the development of adverse reactions in addition to further increasing their bioavailability in the inner ear. This could support the development of randomized clinical trials. Nevertheless, their role in the treatment of autoimmune diseases of the inner ear already considers this treatment modality, and the possibility of their off-label use in clinical research could lead to the valuation of steroids as a potential drug class for the medical treatment of otosclerosis.

##### Nonsteroidal anti-inflammatory drugs

These drugs act by inhibiting the activity of cyclooxygenase, which converts arachidonic acid into prostaglandins. Prostaglandins play a pleiotropic role in bone tissue by inducing both absorption and synthesis.^275^ Indomethacin, a potent representative of this class of drugs, has already been related to inhibition of the bone resorption process in *in vitro* and *in vivo* models.^274^ The role of this drug has yet to be determined. In the absence of studies demonstrating its effect in patients with otosclerosis, its long-term use is not recommended due to the side effects, in addition to the lack of studies in patients with otosclerosis.

##### Possible targeted therapies: immunobiologicals

Immunomodulatory drugs currently in use or approved for use in the country in other chronic inflammatory bone diseases, such as methotrexate, cyclophosphamide, and azathioprine, have not yet been tested in the early stage (or inflammatory stage) of otosclerosis. Therefore, their use is not recommended for otosclerosis due to a lack of evidence in the literature.

As previously described, TNF-alpha is one of the most important pro-inflammatory cytokines and acts by inducing RANKL- and DKK-dependent pathways.^261^ TNF-alpha is synthesized by the otosclerotic focus in the otic capsule in the inflammatory stage of otosclerosis. Therefore, the administration of drugs that inhibit its synthesis/action in the inflammatory stage of otosclerosis may have an effect on the development of the disease.^276^ The use of anti-TNF-alpha agents is validated in the literature for other diseases associated with autoimmunity.

In the field of Otology, few off-label initiatives have been reported for these agents in autoimmune inner ear disease for which conventional steroid therapy provided no benefits to patients. These studies, even with nonsignificant results, point promisingly to favorable outcomes of their use for patients with autoimmune inner ear hearing loss.

Clinically, two strategies can block the effects of TNF-alpha: the use of anti-TNF-alpha antibodies (eg, infliximab) and the use of a recombinant p75 TNF-alpha receptor (etanercept). Both have been tested for the treatment of autoimmune inner ear disease and cochleovestibular disorders, such as Ménière’s disease, with still inconclusive but promising results.^275^, ^277^ Its off-label clinical use can be recommended for the early stage of otosclerosis in randomized clinical trials dedicated to clinical research and will have, in the promising future, an important limitation related to the high added cost of these drugs.

Anti-CD20 antibodies, represented by rituximab, have recently been associated with studies of immune-mediated inner ear disease, with results that are still preliminary but encouraging.^278^ Its use in clinical practice relies on the experience and safety described for use in B-cell lymphoma rheumatoid arthritis. There are no reports for patients with otosclerosis, and its off-label use for clinical investigation purposes should only be recommended in randomized clinical trials.

Other substances and therapy modalities for the inflammatory process have appeared and will continue to appear in the literature with a potentially promising role. In this regard, the anti-RANKL antibody denosumab and the cathepsin K inhibitor odanacatib have been approved for use in patients with severe postmenopausal osteoporosis and may, in the future, have their use expanded to other diseases that affect bone metabolism.

##### Recombinant osteoprotegerin

OPG has an important anti-osteoclastogenic action and acts indirectly by opposing the anti-TNF-alpha and RAK/RANKL actions.^36^ Preliminary studies, not used for clinical practice but in animals, are promising due to the potential action of OPG in the early stage of otosclerotic disease. Therefore, its use should be encouraged in research, initially, only after safety and efficacy have been evaluated in preliminary human studies.^34^, ^279^ Currently not recommended for clinical research pending completion of preclinical studies.

#### Recommendations (Box 14)

**Box 14:** Recommendations for nonsurgical treatment in clinical otosclerosis.

The use of hearing aids is well indicated for the treatment of patients with otosclerosis. However, when compared with stapes surgery, the cost-benefit ratio is worse (Strong recommendation – Low-quality evidence).
Patients with otosclerosis and severe mixed hearing loss with an ABG > 30 dB should consider stapedotomy with subsequent use of a PSAP. Results are good when discrimination of disyllabic words is greater than 50% at 70 dB^280^ (Strong recommendation – Low-quality evidence).
Sodium fluoride has been used for decades to treat patients with otosclerosis. However, well-designed studies are lacking to support its indication (Insufficient evidence).
The use of bisphosphonates has shown radiologic improvement on control scans, but only slight clinical improvement in patients. Higher-quality studies are still lacking to support their indication in patients with otosclerosis (Insufficient evidence).
High-quality studies are lacking to support the indication of vitamin D (Insufficient evidence).

### Implantable systems

The surgery indicated for auditory rehabilitation in patients with otosclerosis is stapedotomy, which is a safe procedure if performed by an experienced surgeon. There are few indications for the use of Active Middle Ear Implants (AMEIs) or Bone-Anchored Hearing Devices (BAHDs) in patients with otosclerosis for two reasons: 1) The disease may progress and worsen the patient’s hearing thresholds, no longer reaching the indication for the use of implants; and 2) Patients usually adapt satisfactorily to the use of conventional hearing AIDS. Therefore, these systems should be indicated only in exceptional cases (Box 15).

**Box 15:** Indications for middle ear implants or bone-anchored hearing devices in patients with otosclerosis.

Eczematous otitis externa that precludes the use of PSAPs or no adequate gain is obtained with the device
Unfavorable surgical anatomy (persistent stapedial artery, obliteration of the oval window by the dehiscent facial nerve)
Otosclerotic *foci* with oval window obliteration
Patients with otosclerotic *foci* in single-sided deafness
Risk of loss of work function due to complications of stapes surgery

#### Bone-anchored hearing devices

Brånemark first demonstrated in 1965 that titanium implants form strong bonds with bone tissue through a process he called “osseointegration”. In 1977, Tjellström inserted titanium implants in the mastoid process in three adult patients with conductive hearing loss and attached a vibrator to the percutaneous implant, being the first to use a hearing aid anchored in the temporal bone. Sound energy is transmitted by skull bone vibrations directly to the cochlea, bypassing the middle ear.^281^

Surgically implanted BAHDs can be divided into percutaneous and transcutaneous. Percutaneous: stimulus occurs via a skin-penetrating abutment coupled to a sound processor – Baha Connect (Cochlear BAS, Gothenburg, Sweden) and the Ponto system (Oticon Medical AB, Askim, Sweden). Transcutaneous BAHDs transmit sound through intact skin, but they can function either actively or passively. Active transcutaneous: an active implant is placed under the skin and muscles of the temporal bone and communicates with the external sound processor wirelessly via radiofrequency – Bonebridge (MED-EL, Innsbruck, Austria) and Osia2 (Cochlear BAS, Gothenburg, Sweden). Passive transcutaneous: a titanium plate is implanted in the temporal bone and a processor is coupled to a magnet that transmits sound through intact skin – Baha Attract (Cochlear BAS, Gothenburg, Sweden) and Sophono (Medtronic, Jacksonville, FL).^282^, ^283^

Patients with disorders that lead to occlusion of the EAC, such as congenital malformations, acquired stenosis of the EAC, and benign tumors, particularly benefit from the use of BAHDs due to the relatively large ABG associated with normal cochlear function.^284^ Because BAHD functioning depends only on bone conduction, these devices have been indicated in some cases of otosclerosis. However, it is not the best option due to the risk of deterioration of the cochlear reserve over the years. Also, stapedotomy complication rates are low with experienced otologists.^132^

Another point to consider when indicating BAHDs is the maximum hearing gain they can offer, considering bone conduction, which varies according to the model, as shown in the box below. In asymmetric hearing loss, BAHDs should be placed only on the side with better bone conduction (Table 9).

**Table 9:** Maximum hearing gain from BAHDs, considering bone conduction.

Model	Maximum gain (dB)**
Ponto (superpower)	65
Baha (superpower)	65
Baha Attract	45
Sophono	45
Bonebridge	45
Osia2	55

** Decibel.

#### Active middle ear implants

AMEIs emerged in the 1990s as a treatment option for patients who could not use a PSAP.^285^, ^286^ They provide functional gain with speech recognition improvement superior to that of PSAPs, with no occlusion effect or feedback for most of them. AMEIs are widely indicated for sensorineural, conductive, or mixed hearing loss. They can be used in middle or outer ear malformations and in advanced otosclerosis. These devices can be fully or partially implantable, depending on the location of the power source and microphone.

AMEIs amplify hearing by mechanically vibrating the ossicles to which they are surgically attached. These devices require movements of the ossicular chain, which are often limited in patients with otosclerosis. They may be indicated in combination with stapedotomy in moderate-to-severe mixed hearing loss or in patients previously subjected to stapes surgery who have developed SNHL^287^ and do not benefit from hearing aids. In advanced otosclerosis, there are 2 implants that have been mostly indicated over time – Vibrant Soundbridge (VSB; Med-El, Innsbruck, Austria) and Codacs (Cochlear Ltd., Sydney, Australia) (Table 10). The latter has been discontinued. Other implantable systems are not indicated in patients with otosclerosis.

**Table 10:** Indicated, contraindicated, or discontinued middle ear implants for patients with otosclerosis.

Device	Indication
Vibrant Soundbridge	Yes
Codacs	Discontinued
Esteem	No
Carina	Discontinued
Maxum	No

##### Vibrant soundbridge

The VSB has 2 components – an external sound processor and an internal component or Vibrating Ossicular Replacement Prosthesis (VORP).^288^, ^289^ The external component is composed of a microphone, audio processor, battery, transmitter, and magnet. It processes acoustic signals into an amplitude-modulated signal and delivers them via electromagnetic waves to the internal component (VORP).^289^ The VORP is composed of a receiver coil, conductor link, and floating mass transducer (FMT).^290^, ^291^

The FMT is the key component of the VSB. It consists of an electromagnetic coil inside a titanium housing that surrounds a small magnet. It is 2.3 mm long, 1.6 mm in diameter and weighs 25 mg.^292^ When the FMT is connected to a moving structure (ossicles or inner ear window), these vibrations can be transferred to stimulate the cochlea.^290^, ^292^

The VSB was developed in the 1990s for patients with SNHL.^292^ Initially, the FMT was coupled only to the long process of the incus through a small embedded titanium clip.^293^, ^294^ Models were manufactured for the right ear or the left ear according to the orientation of the clip. Over time, the FMT was coupled to the round and oval window, which expanded the possibilities of using the VSB for conductive or mixed hearing loss.^289^, ^295^, ^296^

In 2014, VSB model 503 was released, which does not have a titanium clip embedded in the FMT but has a variety of couplers.^292^, ^297^ This model allows the FMT to be placed on the short process of the incus, facilitating surgery. Other couplers were developed both for the round window and to aid ossiculoplasty, and they can also be adapted to a partial or total ossicular replacement prosthesis in middle ear surgery.^292^, ^298^

Coupling the FMT to the short process of the incus,^289^, ^292^ requiring only mastoidectomy and atticotomy, with no need for posterior tympanotomy, reduced operative time and risks.^298^ Studies have shown increased amplification compared with other coupling options.^292^, ^299^

Another indication is coupling the VSB to the round window after subtotal petrosectomy in patients with extensive cholesteatoma or neoplasms that have caused extensive destruction of the middle ear.^300^, ^301^

After surgery, it is usually unknown whether the FMT is functioning properly. Currently, hearing results can be tested by brainstem evoked response audiometry with a chirp stimulus through the FMT after the end of the procedure.

The VSB may be indicated in patients with otosclerosis after the hearing loss has been stable for more than 12 months. It is essential to inform the patient of the risks of hearing loss over time or during the surgical procedure, reducing the functional gain with the device.^302^, ^303^

The VSB can be placed on the short process of the incus in patients with conductive hearing loss who do not adapt to or gain little benefit from a PSAP and are unwilling to accept the risks of stapedotomy (PSA or complete obliteration of the oval window by the facial nerve). In patients with moderate/severe mixed hearing loss, VSB can also be used in combination with stapedotomy.^302^

The FMT can be placed on the round window, which is facilitated by the use of specific couplers.^292^ However, there is a risk of creating a round window opening during surgery, which may lead to deafness,^304^ in addition to the risk of otosclerotic *foci* in the round window,^305^ a contraindication to the procedure. This is indicated when coupling to the incus is not possible due to erosion of the long process. It may be an alternative to malleostapedotomy.

##### Codacs

In 2008, Hausler et al. presented an implantable hearing system that included a newly developed transducer, the Direct Acoustic Cochlear Stimulator (DACS).^306^ It directly stimulates the inner ear by vibrating the perilymph. Stimulation of the perilymph occurs via a conventional stapes prosthesis. The device consisted of the transducer, a fixation system, and a percutaneous plug, to which an externally worn sound processor was connected.^307^ It was implanted in 4 patients with severe-to-profound mixed hearing loss during a clinical trial. Based on the DACS concept, the trial showed that the hearing and speech intelligibility of the patients improved after implantation compared with the preoperative condition.^306^

The Cochlear Nucleus Freedom sound processor (Cochlear Ltd., Sydney, Australia) was adapted to deliver acoustic information to the implantable electronic system by using specific software, being then called Codacs.^306^, ^307^, ^308^ It is indicated in adults with severe-to-profound mixed hearing loss caused by advanced otosclerosis.^309^ The device is incompatible with MRI.

The Codacs external device consists of a behind-the-ear sound processor that communicates via radiofrequency with the internal device. The implantable part consists of a receiver coil, the implant electronics, and the electromagnetic transducer. Sound is picked up by the sound processor’s microphone and converted into a digital signal, which is then broken down into its constituent frequency components (20 bands), amplified, and processed. The processed sound is then transmitted according to parameters similar to those of the Cochlear Nucleus Freedom implant (Cochlear Ltd.) with high-rate protocol.^308^

The implant decodes the signal and sends a stimulating current to the electromagnetic transducer. The transducer vibrates the stapes prosthesis, thereby mechanically stimulating the perilymph of the inner ear. Codacs was used in other applications, particularly in patients with ossicular chain disruption (after cholesteatoma, infections, or surgical manipulation).

The ossicular chain should be manipulated for removal of the long process of the incus and the stapes superstructure using a laser. Once a good position for the stimulator is found, the device is fixed in place. A conventional stapes prosthesis is inserted into the footplate perforation and crimped to the long process of the artificial incus of the actuator.

A European multicenter trial included 15 patients with advanced otosclerosis and severe bilateral mixed hearing loss. Despite the short follow-up (3 months), pure-tone thresholds did not worsen after the procedure and the mean improvement in pure-tone thresholds was 48 dB (all frequencies showed a statistically significant improvement postoperatively), with improvement in the speech intelligibility index.^307^

A retrospective study compared 25 patients with Codacs (≥3 months after activation) with 54 patients using CIs (2 years after activation) with comparable preoperative bone conduction thresholds.^310^ Speech intelligibility in noise was significantly better in patients with Codacs (median 80%) than in patients with CIs (median 25%). This device is currently discontinued.

#### Cochlear implantation

SNHL in patients with otosclerosis occurs when ionic homeostasis of the cochlea is disrupted due to atrophy and hyalinization in the stria vascularis and spiral ligament. Consequently, dysfunction or loss of HCs and loss of spiral ganglion can occur.^311^

Approximately 10% of patients with otosclerosis and conductive hearing loss also develop SNHL.^312^ Advanced otosclerosis is characterized by SNHL and decreased speech discrimination (<30% at 70 dB),^280^ associated with radiologic abnormalities.^313^

The severity of SNHL in otosclerosis is correlated with radiologic abnormalities on HRCT, which can detect oval window abnormalities in 80%–90% of cases.^97^, ^314^ On CT, the finding of pericochlear lucencies is highly specific for otosclerosis. It presents as a double halo.^96^, ^315^ T1-weighted MRI images may show a ring of intermediate signal in the pericochlear area with mild-to-moderate gadolinium enhancement.^316^ T2-weighted sequences are the best method to assess the patency of the cochlear duct.^97^, ^317^

There is a consensus in the literature regarding the indication of cochlear implantation as a safe and beneficial treatment in cases of advanced otosclerosis.^314^, ^318^, ^319^ Recent systematic reviews on advanced otosclerosis reported that patients undergoing CI surgery experienced no major surgical complications.^318^, ^320^, ^321^ Despite technological advances in cochlear implantation in recent decades, otosclerosis presents unique challenges.

Intraoperative difficulties include ossification, partial obliteration of the basal turn and round window, and false tract insertion of electrode array into the cochlea.^322^ In a case series of advanced otosclerosis treated with CI surgery, the round window membrane was ossified in 60% of cases and the scala tympani in 30% of cases.^323^ Recently, software programs have been developed by CI manufacturers with the purpose of conducting a detailed planning of the surgery, identifying the best electrode array system, and choosing the best electrode insertion method based on CT data.^324^, ^325^

Regardless of complications with electrode insertion, otosclerosis is an etiologic factor negatively correlated with the speech performance scores of CI users.^326^ Remodeling of the otic capsule alters the properties of electric current conduction in the cochlea, which may impair CI use over time. Increased electrode impedance^327^ and facial nerve stimulation, causing paresthesia, muscle spasms, and pain,^96^, ^312^, ^315^, ^327^ require changes in the device programming and stimulation strategies.^97^, ^317^ In view of such findings, more frequent mapping adjustments are recommended to adjust and optimize the stimulation parameters.^314^, ^320^, ^322^, ^328^

Studies indicate increases, although not significant, in the minimum and maximum electrical stimulation levels (T and C levels) in patients with advanced otosclerosis compared with other etiologies.^320^, ^328^ The progression of otosclerotic *foci* often occurs in the basal and medial regions of the cochlea and, due to decreased impedance of the otic capsule and flow of the electric current through the bone, the electric current required to stimulate the fibers of the auditory nerve is increased. Mapping adjustments are essential to manage this situation. With the increase in stimulation levels, if the perceived intensity is not adequate, there is a need to increase pulse duration, which can potentially result in a decrease in stimulation frequency and compromise the proper functioning of the chosen processing strategy. In certain situations, there may be a need to switch off the electrodes to avoid the negative effects generated by the significant increase in stimulation levels.^314^, ^329^, ^330^

Facial nerve stimulation resulting from a shunt of current from the otic capsule that reaches the labyrinthine segment of the facial nerve^96^, ^319^, ^329^ has been described as one of the most common postoperative complications in patients with advanced otosclerosis, with an average incidence of 20% in this population, reaching up to 75%.^96^, ^320^, ^331^ Authors have suggested that the high incidence of facial nerve stimulation is associated with the type of electrode array used (straight or perimodiolar), with the straight or more distal array showing a higher incidence.^96^, ^314^, ^328^, ^331^

Facial nerve stimulation can occur both at the time of electrode activation and during subsequent device monitoring visits.^318^ To eliminate or at least minimize its effects, mapping adjustments must be done, such as decreasing the electric charges by changing the stimulation mode ‒ by reducing the amplitude of the maximum current levels, keeping them below the stimulation threshold for the facial nerve, or even by adjusting the biphasic pulse width.^330^ More recently, triphasic pulse patterns have also been successfully used to alleviate the symptoms of facial nerve stimulation.^327^, ^329^

Switching off the problematic electrodes has been another procedure frequently described in studies as an alternative method to manage facial nerve stimulation.^321^ There is no consensus on which electrodes (basal, medial, or apical turn electrodes) are the most affected in facial nerve stimulation.^96^, ^331^ The fact is that, depending on the number of deactivated electrodes, speech perception can be significantly affected. In these cases, reoperation using another CI model with characteristics suitable for targeted electrical stimulation (related to the positions of intracochlear electrode contacts, electrode geometry, and stimulation parameters) as well as sequential cochlear implantation may be viable alternatives to be considered.^320^, ^330^, ^331^

Most studies point to promising auditory perception outcomes in patients with advanced otosclerosis. Numerous studies have not found significant differences in word and speech recognition performance scores between implanted patients with advanced otosclerosis and those with other etiologies.^318^, ^320^, ^330^ In fact, it is assumed that this disease has little effect on the spiral ganglions of the auditory nerve.^324^ However, although not significant, some authors have reported a trend toward poorer performance in the group with advanced otosclerosis. The poorer results obtained in patients with advanced otosclerosis were associated with factors such as long deafness periods, older age, extensive ossification, presence of facial nerve stimulation, and a greater number of deactivated electrodes.^318^, ^319^, ^328^

#### Recommendations (Box 16)

**Box 16:** Recommendations for implantable systems in otoclerosis patients.

Before indicating BAHDs or AMEIs in patients with otosclerosis, hearing devices should be tested (Strong recommendation – Low-quality evidence).
Patients with an ABG > 30 dB, conductive hearing loss, and contralateral ear with deafness benefit from the use of BAHDs (Moderate recommendation – High-quality evidence).
Patients should have a stable audiogram for more than 2 years before BAHD or AMEI is indicated (Strong recommendation – Low-quality evidence).
Indications for cochlear implantation in patients with advanced otosclerosis are the same as those in patients with other causes of profound deafness – (1) Speech intelligibility index ≤60% for open-set speech recognition with the use of a PSAP in the better ear and ≤ 50% in the ear to be implanted; (2) Psychological suitability and motivation for CI use, for CI maintenance/care, and for the speech rehabilitation process; (3) Appropriate rehabilitation conditions in the city of origin (referral/counter-referral); (4) Commitment to care for the external components of the CI and to attend the speech rehabilitation sessions (Strong recommendation – High-quality evidence).
Patients with advanced otosclerosis are at increased risk of ossification of the round window membrane and basal turn, and the surgeon should order MRI as a mandatory test to prevent complications during the insertion of electrode array (Strong recommendation – Moderate-quality evidence).
The use of perimodiolar electrodes reduces the risk of facial nerve stimulation compared with lateral wall electrodes (Moderate recommendation – High-quality evidence).

## Conclusions

The pathophysiology of otosclerosis has not yet been fully elucidated, but environmental factors and unidentified genes are likely to play a significant role in it. Women with otosclerosis are not at increased risk of worsening clinical condition due to the use of contraceptives or during pregnancy. Drug treatment has shown little benefit. If the patient does not want to undergo stapedotomy, the use of hearing aids is well indicated. Implantable systems should be indicated only in rare cases, and the CI should be indicated in cases of profound deafness.

## Final recommendations (Box 17)

**Box 17:** Recommendations for otosclerosis.

Despite evidence of a genetic origin of otosclerosis, there is currently no indication for genetic testing in patients with otosclerosis due to gene variability, poorly understood modes of inheritance, and a not fully elucidated role of identified factors (Strong recommendation – Low-quality evidence).
There is more favorable evidence linking otosclerosis to measles virus infection, also evidenced by the reduction in cases of otosclerosis after measles vaccination over the years. However, measles vaccination should not be recommended as a good public health policy to reduce the number otosclerosis cases (Insufficient evidence).
There is no evidence that pregnancy or the use of oral contraceptives increases the risk of developing or worsening otosclerosis (Strong recommendation – High-quality evidence).
Patients with conductive hearing loss, with Carhart notch on the audiogram, absence of stapedial reflex, type Ar tympanogram, family history of otosclerosis, and successful surgery in one of the ears gain little benefit from imaging (Moderate recommendation – Low-quality of evidence).
Patients who are candidates for stapedotomy should undergo vestibular evaluation (pre or postoperative) (Insufficient evidence).
Stapes surgery is recommended for patients with conductive hearing loss with mean pure tone thresholds ≥25 dB at 250 Hz, 500 Hz, 1000 Hz, and 2000 Hz and an ABG ≥ 20 dB (Strong recommendation – Moderate-quality evidence).
Revision surgery is not indicated for SNHL (Strong recommendation – Moderate-quality evidence).
Among nonsurgical treatment options, hearing devices provide the best result. Drug treatment has shown few satisfactory results (Strong recommendation – Low-quality evidence).
Except for the CI, implantable systems are not very suitable for the treatment of otosclerosis and should be indicated only in rare cases (Strong recommendation – Low-quality evidence).

## Conflicts of interest

The authors declare no conflicts of interest.

This research did not receive any specific grant from funding agencies in the public, commercial, or not-for-profit sectors.
